# High-Throughput Single-Cell Derived Sphere Formation for Cancer Stem-Like Cell Identification and Analysis

**DOI:** 10.1038/srep27301

**Published:** 2016-06-13

**Authors:** Yu-Chih Chen, Patrick N. Ingram, Shamileh Fouladdel, Sean P. McDermott, Ebrahim Azizi, Max S. Wicha, Euisik Yoon

**Affiliations:** 1 Department of Electrical Engineering and Computer Science, University of Michigan, 1301 Beal Avenue, Ann Arbor, MI 48109-2122, USA; 2 University of Michigan Comprehensive Cancer Center, 1500 East Medical Center Drive, Ann Arbor, MI 48109-5940, USA; 3 Department of Biomedical Engineering, University of Michigan, 2200 Bonisteel Blvd, Ann Arbor, MI 48109-2099, USA

## Abstract

Considerable evidence suggests that many malignancies are driven by a cellular compartment that displays stem cell properties. Cancer stem-like cells (CSCs) can be identified by expression of cell surface markers or enzymatic activity, but these methods are limited by phenotypic heterogeneity and plasticity of CSCs. An alternative phenotypic methodology based on *in-vitro* sphere formation has been developed, but it is typically labor-intensive and low-throughput. In this work, we present a 1,024-microchamber microfluidic platform for single-cell derived sphere formation. Utilizing a hydrodynamic capturing scheme, more than 70% of the microchambers capture only one cell, allowing for monitoring of sphere formation from heterogeneous cancer cell populations for identification of CSCs. Single-cell derived spheres can be retrieved and dissociated for single-cell analysis using a custom 96-gene panel to probe heterogeneity within the clonal CSC spheres. This microfluidic platform provides reliable and high-throughput sphere formation for CSC identification and downstream clonal analysis.

There is now substantial evidence that many cancers are heterogeneous and hierarchically organized, and that at the apex of this hierarchy are cells that display stem cell properties. These cancer stem-like cells (CSCs) drive tumor growth and metastasis and contribute to treatment resistance^1^,^2^,^3^,^4^,^5^,^6^,^7^. This suggest that more effective cancer therapies will need to target the CSC population, rather than simply reducing overall tumor burden^3^,^8^,^9^,^10^. This presents a problem as this heterogeneity has been challenging to study. Although the identification of CSCs via surface and enzymatic markers has been useful, the phenotypic heterogeneity and cellular plasticity of CSCs limits their use^11^,^12^. This highlights the need for functional CSC assays which characterize diverse CSC populations.

First utilized for the identification of neural stem cells, *in vitro* sphere formation assays have also been suggested as a marker free methodology for culture and identification of stem-like cells in breast and other cancers^13^,^14^,^15^. At the most basic level, these are anoikis-based assays. For normal differentiated cells, adhesion to an extracellular matrix (ECM) scaffold is essential for maintenance of cellular homeostasis; disruption of cell attachment leads to anoikis, a form of programmed cell death^16^. Stem cells have the ability to survive in anchorage-independent conditions, likely mediated by constitutive activation of focal adhesion kinase (FAK) in these cells^13^. When breast cancer cells are cultured in suspension, bulk non-stem cells undergo anoikis, while only stem-like cells survive and proliferate to form spheres, as they are anoikis resistant^13^. As such, the formation of mammospheres from normal mammary stem cells or tumorspheres from breast cancer stem cells can be used to identify cells with these stem-like characteristics.

In practice, there are a number of issues limiting the utility of these assays^17^. For proper selection of single-cell derived spheres, cell aggregation must be prevented, so that anchorage dependent cells cannot adhere together to survive and proliferate. When using conventional culture methods such as dishes or plates, cell-seeding density must be carefully controlled. Even with proper methodology it has been reported that many mammospheres are not single-cell derived, but in fact, aggregation of the seeded cells^18^,^19^. Although anti-aggregation additives (e.g. Heparin) can be used, these may affect cell behavior^20^. In addition, reliable media exchange can also be problematic. Cells can be easily lost or disrupted when replacing the media and early spheres can be dissociated; as a result, the duration of the assay is limited by nutrient depletion and waste buildup. Finally, studies performed in neurospheres suggest intermediate progenitor cells may also form spheres, but with different initiation and proliferation rates. Sphere formation rates might thus overestimate actual sphere forming frequency^21^,^22^. Robust high throughput single-cell derived sphere formation, tracking, and downstream sphere analysis are needed to identify and study potential CSCs in tumorsphere assays. A microfluidic approach is ideally suited to address these needs.

There are a number of microfluidic approaches for performing sphere assays on-chip, including hanging droplet methods^23^,^24^,^25^, micro-rotation flow^26^, and micro-well^27^. These platforms are generally not clonal, and thus they do not exclude the possibility of cellular aggregation. As such, these platforms are not suitable for performing tumorsphere/CSC assays. While droplet-based systems can isolate single cells in suspension, it is difficult to continuously provide fresh media to each droplet, preventing long-term culture^28^. Recently, we demonstrated successful suspension cell culture in our single-cell platform^29^,^30^ by integrating topographically-patterned polydimethylsiloxane (PDMS) layers to provide a super-hydrophobic surface for facilitating suspension cell culture^31^. Despite its advantages over many conventional suspension culture coatings and devices, the patterned surface requires expensive deep reactive ion etching and complicates optical imaging.

In this work, we report a scalable single-cell suspension culture chip with 1,024 micro-chambers with non-adherent surface coating, which can provide robust single-cell isolation, tracking, and continuous media perfusion to avoid any difficulty in cell seeding and media exchange. The sphere formation potential of multiple cell lines and primary patient derived xenographs (PDX) were compared and assessed. We also investigated the relationship between sphere formation and expression of genes related to cell ‘stemness’. Finally, we demonstrated the ability to retrieve and dissociate the single-cell derived spheres and performed multiplexed single cell pCR mRNA analyses to ascertain the degree of cellular heterogeneity within clonally derived spheres.

## Results

### Single cell capture scheme

**Figure 1:**
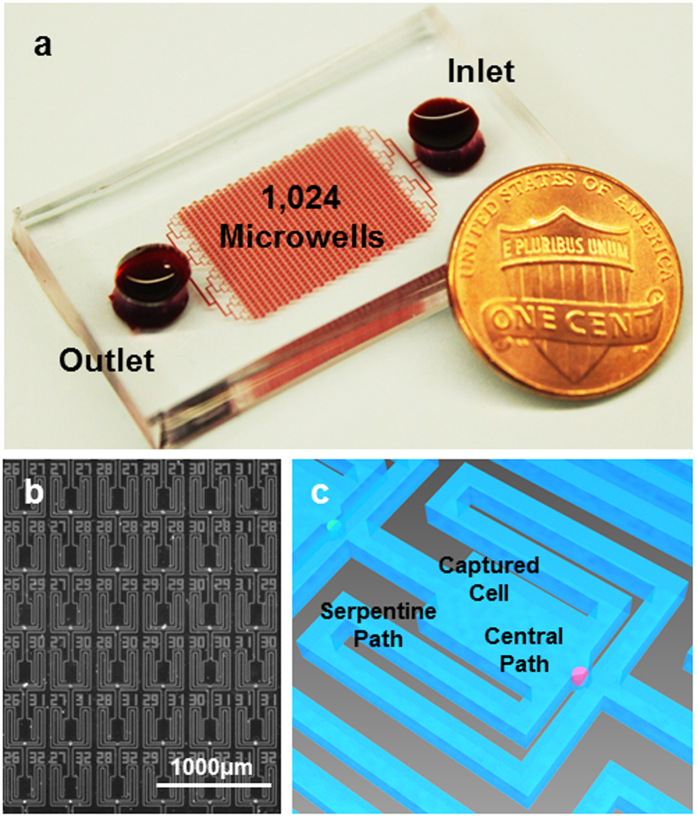
Microfluidic chip for single cell sphere formation. (**a**) Photograph of the fabricated 1,024-microchamber device, in which the media containing cells flows from the inlet to the outlet by gravity flow induced from the difference in media heights between the inlet and outlet, simplifying single cell deployment at high capture rate for various cell lines and primary samples without using an external pump. (**b**) Micrograph of captured MDA-MB-231 cells in the fabricated chip, showing a 6 by 6 segment with 89% capture rate. (scale bar: 1000 μm). (**c**) Enlarged schematic of a cell capture chamber with a central path and serpentine path.

To develop a microfluidic chip for tumorsphere culture and analysis, we adapted our previous single-cell hydrodynamic capture scheme^29^. This scheme is fabricated with a standard PDMS soft lithographic process molded from a two layer SU-8 master (Supplementary Fig. 1). Figure 1a shows the fabricated device with 1,024 micro-chambers and integrated non-adherent coating. Cells are loaded from the inlet by gravity flow that is generated spontaneously due to a liquid height difference between the inlet and the outlet. No external pumps are used for cell loading or culture. In each micro-chamber, there are two fluidic paths: a central path and a serpentine path (Fig. 1c). The central path has lower flow resistance, so the first cell entering the chamber tends to flow through the central path (Supplementary Fig. 2, Supplementary Movie 1). As the opening of the central path is smaller than the cell size (10 μm by 15 μm in our design), it sterically captures the cell. The captured cell will thus block the central path, increasing the central path resistance. Following cells will then preferentially flow through the serpentine path and be captured in the downstream micro-chambers (Supplementary Fig. 2f, Supplementary Movie 2). Cell capture experiments have been performed with various cell types, and high cell capture rates (>70%) have been achieved in all of these cell lines (Supplementary Table 1). Figure 1b shows an indexed 6 by 6 subset of the array of culture chambers, in which 32 single MDA-MB-231 cells were captured (89% capture rate). The captured single cells can be easily tracked through optical imaging, making this platform ideal for quantifying heterogeneous cell populations and for applications where single cell seeding is critical. Using this chip, we can capture, suspension culture, and monitor nearly 700–800 single cells in a single experiment, which outperforms previous suspension culture platforms in throughput and capture rate^32^,^33^.

### Characterization of integrated polyHEMA coating

**Figure 2:**
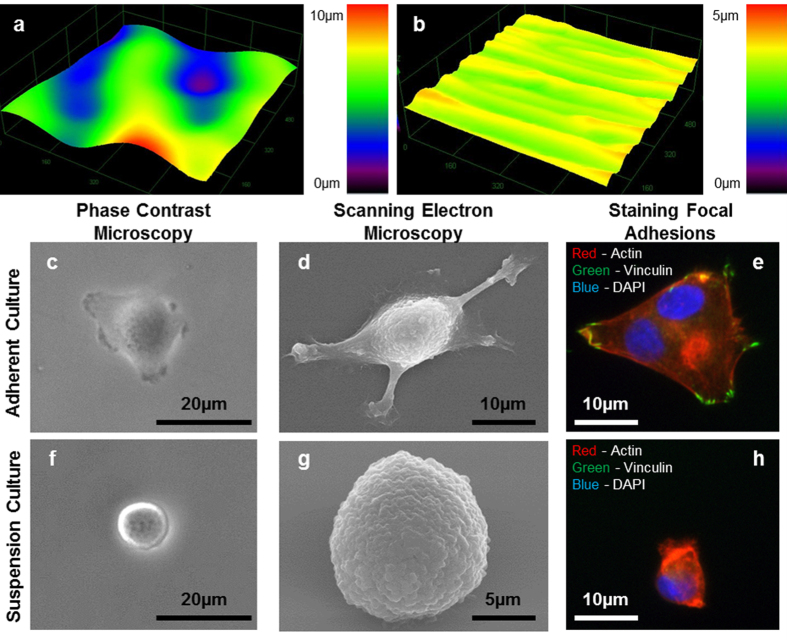
Surface properties of polyHEMA films. 3D surface profile of polyHEMA coated substrate by (**a**) conventional evaporation process or (**b**) by spin coating and reflow process. MDA-MB-231 cells were cultured for 24 hours on (**c–e**) non-coated glass substrate or (**f–h**) polyHEMA coated substrate. Phase contrast micrographs (**c,f**) and scanning electron micrographs (**d,g**) of single MDA-MB-231 cells. (**e,h**) MDA-MB-231 cells were stained for actin (red), vinculin (green), and nucleus (DAPI blue).

To achieve suspension culture in our single-cell platform, we optimized a poly 2-hydroxyethyl methacrylate (polyHEMA) hydrogel coating process to form non-adherent microchambers^34^,^35^,^36^. Conventional polyHEMA coating techniques create rough, non-uniform surfaces (Fig. 2a) that require thick layers for robust suspension culture. In order to overcome these issues, we developed an alternative spin coating and reflow process that improves roughness and uniformity (Fig. 2b)^37^. The surface roughness root mean square (RMS) of the conventional coating process is greater than 3 μm, while that of the spin-coated films is less than 0.2 μm (Supplementary Table 2). The thin and uniform spin-coated polyHEMA films can reliably bond with the PDMS after oxygen plasma activation, creating a robust non-adherent culture environment (Supplementary Fig. 3).

### Suspension culture on polyHEMA-coated substrate

After characterizing coating quality, suspension cell culture was performed on open coated substrate (polystyrene dish with polyHEMA coating) to be compared with culture on uncoated surfaces (polystyrene dish alone). MDA-MB-231 breast cancer cells cultured for 24 hours without polyHEMA coating exhibit spreading morphology under both bright-field microscopy and scanning electron microscopy (Fig. 2c,d). In contrast, MDA-MB-231 cells cultured on the polyHEMA-coated surfaces did not appear to form focal adhesions and cells maintained a rounded morphology (Fig. 2f,g). Focal adhesions serve as the link between internal cytoskeletal networks and the ECM. Therefore, the absence of focal adhesions can be a good indicator whether a cell is truly cultured in a suspension environment^38^. MDA-MB-231 cells were plated on standard tissue culture plastic with and without polyHEMA coatings, and their focal adhesions were stained. Cells cultured on a normal tissue culture plastic expressed distinct vinculin focal adhesion clusters (Fig. 2e), while cells cultured on polyHEMA-coated surfaces did not stain for vinculin (Fig. 2h). No adherent cells or cells with distinct extracellular vinculin clusters were observed in the polyHEMA-coated dishes, confirming a robust non-adherent environment for sphere formation. While other have reported the use of uncoated PDMS surfaces to achieve non-adherent cell culture^39^,^40^, this phenomena is decidedly cell specific. Cancer cells of various types, including T47D (Luminal A breast cancer), readily attach and spread on unmodified PDMS surfaces (Supplementary Fig. 4), supporting the need to develop polyHEMA coating.

### Single-cell derived sphere formation

Single-cell derived sphere formation has been suggested as a surrogate assay for stem cell activity^13^,^14^,^15^,^41^,^42^, but such assays often suffer from poor single cell seeding and control and low throughput as a result of the rarity of CSC. Our platform automates single-cell capture and sphere formation on a large scale (1,024 single-cell microchambers), overcoming many of the issues encountered when performing such assays with conventional techniques. To the best of our knowledge, no other microfluidic chip has demonstrated successful clonal (single-cell derived) sphere formation to date.

**Figure 3:**
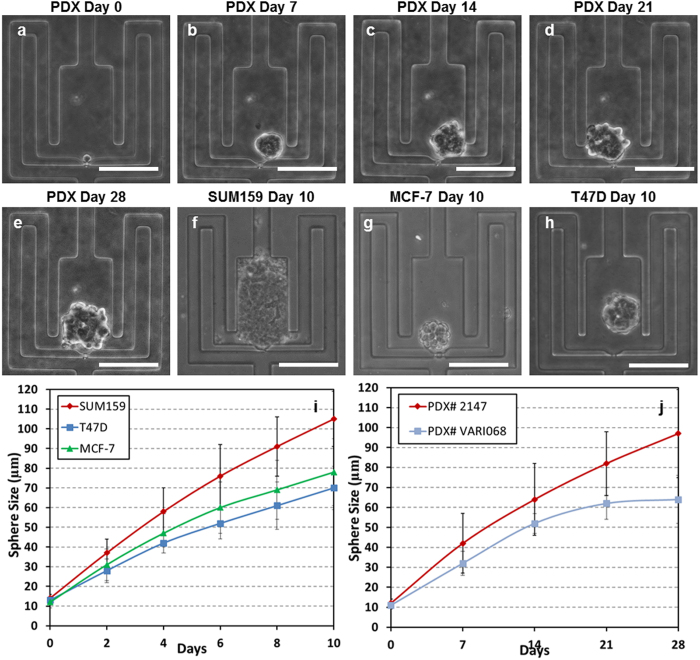
Formation and morphology of cancer spheres derived from a single cell. (**a–e**) The development of a patient derived xenograft (PDX) sphere from a single cell in the polyHEMA-coated suspension culture micro-chamber: (**a**) day 0, (**b**) day 7, (**c**) day 14, (**d**) day 21, (**e**) day 28. (**f–h**) The development of single-cell derived sphere formation for various cell lines: (**f**) SUM159 after 10 days, (**g**) MCF-7 after 10 days, and (**h**) T47D after 10 days. (**i**) The growth (diameter) of SUM159, T47D, and MCF-7 spheres from day 0 to day 10. (**j**) The growth (diameter) of PDX samples over 28-day period. Scale bar: 100 μm.

One complicating factor for tumorsphere assays is the fact that non-stem progenitor cells may also form spheres. The SUM159 claudin-low breast cancer line contains a large pool of such progenitors and, as such, was selected here as a test cell line with high sphere forming potential. Cells were loaded on chip and cultured in serum-free media for 10 days. After loading, SUM159 cells successfully formed spheres (Table 1); 55% of single SUM159 cells could grow to a sphere with a size larger than 50 μm diameter within 10 days (Fig. 3). For images of on chip SUM159 single-cell derived sphere formation, see Supplementary Fig. 5. Additional cell lines, for which tumorsphere assays more accurately reflect the CSC potential, were next tested. Compared to SUM159, MCF-7 and T47D (Luminal A breast cancer) cells have lower sphere-forming potential (Table 1) and result in overall smaller spheres (Fig. 3f–i). Around 2–3% of the captured MCF-7 and T47D cells form spheres. The sphere formation potential of HCC38, MDA-MB-231, and SUM149 were also assessed and matched well with expected CSC potential (Table 1).

In addition to cell lines, single cells from breast cancer patient derived xenografts (PDX) were tested in the platform. PDXs conserve original tumor heterogeneity, molecular signals, and phenotypes, making them ideal for our application^43^. PDXs successfully formed spheres on chip (Fig. 3a–e), showing a slightly lower sphere formation rate as compared to the tested cell lines (Table 1). Though slower growing, PDXs were easy to culture for a longer period in our device to observe sphere formation. With definite single sphere spatial confinement and continuous media perfusion supplied by the gravity flow, the PDXs were continuously monitored over 28 days (Fig. 3j). To demonstrate the general utility of the device, 3 additional non-breast cancer cell lines (A549 human alveolar adenocarcinoma, SKOV3 human ovary adenocarcinoma, and C6 rat glioma) were assayed on chip to assess their sphere formation potential (Supplementary Table 3). These experiments demonstrate the capacity to perform high-throughput and robust single-cell sphere formation assays with a wide variety of cell types and even models of primary breast cancer cells.

### Sphere formation rate of stem-like Notch+ and non-stem-like Notch- cells

**Figure 4:**
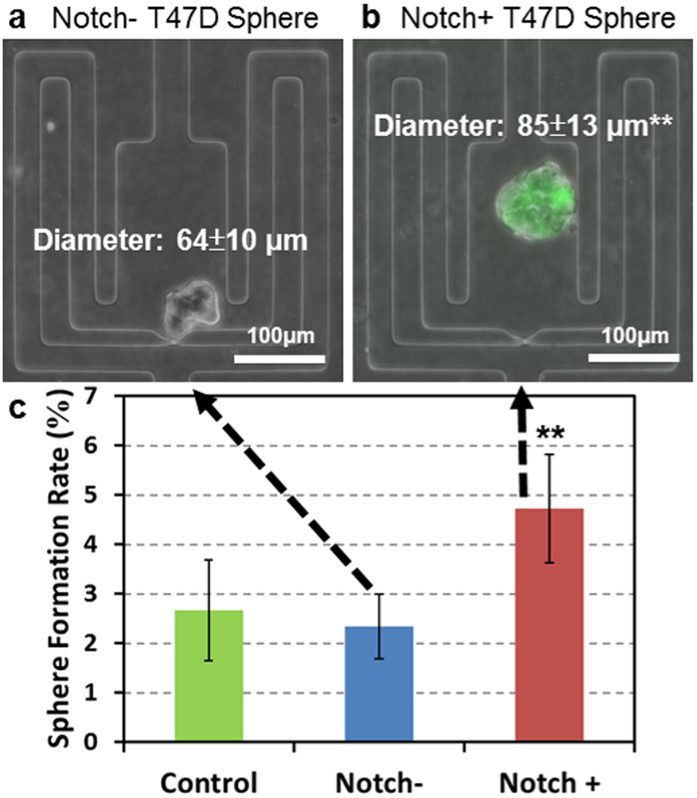
Differential cancer sphere formation from Notch+ and Notch- T47D cells. Representative cancer sphere derived from a single (**a**) T47D Notch- (GFP-) or (**b**) T47D Notch+ (GFP+) cell in the polyHEMA coated suspension culture micro-chamber. (**c**) Sphere formation rate of Notch+ and Notch- cells after 14-day culture. Data are shown as the mean ± SD (N = 4). **refers to P < 0.01.

There are considerable similarities between healthy stem cells and CSCs. In particular, canonical stem cell pathways such as Wnt and Notch have been shown to be critical for the regulation CSC behaviors (e.g. survival and self-renewal)^44^. In fact, high Notch expressing cells have been shown to possess higher tumor initiating potential than cells expressing low levels of Notch^45^,^46^. We therefore examined the relationship between notch pathway activation and sphere formation potential. To monitor Notch pathway activation, we transduced T47D cells with a lentiviral (pGreenFire1) Notch reporter containing multiple Notch response elements upstream of a minimal CMV promoter regulating destabilized GFP. After selection and culture we found that Notch+ cells generate both Notch+ and Notch- daughter cells in culture due to asymmetric division; In contrast Notch- cells produce exclusively Notch- daughter cells. To measure single-cell sphere formation from Notch+ and Notch- T47D cells, we loaded unsorted cells into suspension microfluidic chips and cultured them in serum-free media. Despite the heterogeneous population, no pre-sorting is necessary due to the robust single-cell capture and large throughput of the platform. Although, both Notch+ and Notch- T47D cells formed spheres (Fig. 4a,b), the sphere formation rate and average size of Notch+ cells was significantly higher (Fig. 4a–c). Additionally, spheres formed from Notch- cells contained no Notch+ cells, while spheres formed from Notch+ cells contained both. The small, less frequent notch- spheres generated from notch- cells suggests that these cells may be progenitors rather than true CSC’s. This leads to the need for our assay (and tumorsphere assays in general) to incorporate further downstream analysis of CSC. Genetic expression analysis of the spheres via the Fluidigm C1/Biomark HD system could provide a way to differentiate progenitor-derived spheres and true stem-like cell derived spheres.

### Sphere retrieval and single cell gene expression analysis

Our novel fabrication scheme can facilitate simple sphere retrieval after sphere formation assays have been completed. The bond strength between polyHEMA and PDMS is strong enough to provide a good fluidic seal, but still weak enough to make it reversible unlike PDMS-PDMS and PDMS-glass bonding, which are irreversible. Consequently, to retrieve the spheres, the top layer can be easily peeled off and washed over a culture dish, releasing the spheres inside. An example of a retrieved sphere is shown in Supplementary Fig. 6. At the time of sphere retrieval, there is no risk of non-sphere forming cells contaminating the retrieved sample as the non-adherent environment has induced anoikis and cell death. After anoikis, the remnants of non-sphere forming cells are washed out of the arrays by the gentle perfusion flow. After release, the captured spheres can be deployed into non-adherent 384-well plates for further growth, because the microchamber dimensions on the chip limit the sphere size. Continued growth of the spheres is necessary to provide a larger pool of cells for use with the Fluidigm C1/Biomark HD system for multiplexed single cell genetic expression analysis.

**Figure 5:**
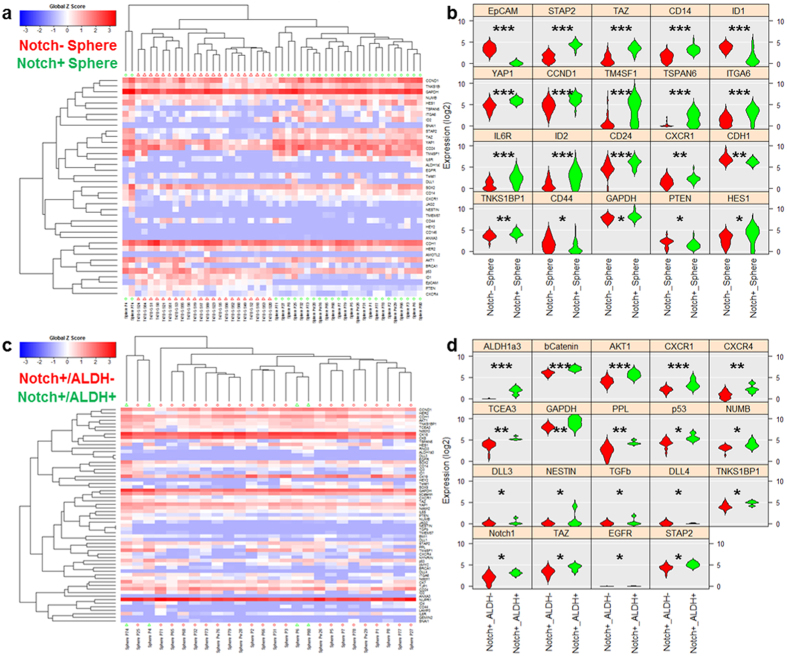
Single cell gene-expression data of retrieved spheres utilizing Fluidigm C1/Biomark HD for multiplexed gene expression analysis. (**a**) Heatmap hierarchical clustering of single cell expression analysis for Notch+ derived and Notch- derived spheres. (**b**) Violin plots of genes that were scored as statistically different between Notch+ derived and Notch- derived spheres (**c**) Heatmap hierarchical clustering of single cell expression analysis for ALDH1a3 high Notch+ derived (N=4) and ALDH1a3 low Notch+ derived cells (N = 22). (**d**) Violin plots of genes that were scored as statistically distinctive between ALDH1a3 high Notch+ derived and ALDH1a3 low Notch+ derived cells. *refers to P < 0.05, **refers to P < 0.01, and ***refers to P < 0.001.

In order to perform Notch+ and Notch- sphere comparison studies, single-cell derived spheres were formed on-chip, released and redeployed in a 384-well plate, and grown to a size of approximately 200 cells. Two spheres of interest (one Notch+ derived and one Notch- derived) were dissociated into single cells via trypsinization and mechanical force (pipetting) and loaded into the Fluidigm C1/Biomark HD system for multiplexed gene expression analysis. CSC genetic signature data from numerous breast cancer cell lines and primary breast cancer samples was used to identify a 96-gene panel that is capable of clustering CSCs from non-stem cells^44^ and was used for analysis herein. Due to number (96) of capture wells, the Fluidigm C1 chip does not capture every single cell that is loaded, so the data generated does not show each cell in the sphere. However, as shown in Supplement Fig. 7, the C1/BioMark HD platform does not have significant bias or preference toward certain population of single cells based on size or morphology, likely allowing capture of a reasonably representative sample. This is further supported by Supplementary Fig. 8, showing the good correlation between the average of single T47D cell (n = 28) expression data and bulk T47D (5,000 cells each sample, n = 3) expression Single cell expression data for the genes that are statistically distinct between the two spheres (one Notch+ and one Notch-) is shown in heatmap and violin plots (Fig. 5a,b). P-values, based on one-way ANOVA, are shown in Supplementary Table 4. (Supplementary Fig. 9). Notch+ and Notch– cells from bulk cultured on adherent surfaces also show a similar principal component analysis (PCA) clustering, so observed effects cannot be attributed to sphere culture alone (Supplementary Fig. 10).

Though dissociated from a clonal sphere, there was also heterogeneity in the form of a small population of aldehyde dehydrogenase (ALDH) isoform 1a3 high-expressing cells within the Notch+ derived sphere cells. When analyzed separately, these ALDH1a3 high cells show markedly different expression of genes (e.g. β-catenin, CXCR1, CXCR4, and p53) (Fig. 5c,d). P-values for the significant genes can been seen in Supplementary Table 5. PCA clustering and the results from the entire gene panel are shown in Supplementary Fig. 11. These cells represent a rare sub-population that would have been ignored in non-single cell-based approaches. When separating solely based on ALDH1a3 expression, only a single Notch- derived cell was also scored as ALDH1a3 high (Supplementary Fig. 12), suggesting that Notch- derived spheres might have decreased “stemness” (1 out of 22 cells) as compared to the Notch+ derived spheres (4 out of 26 cells).

## Discussion

We report the development of microfluidic platform for single-cell suspension culture using a hydrodynamic capture scheme and optimized polyHEMA coating technique for the performance of robust and high-throughput tumorsphere assays. Cell loading provides robust and reproducible single-cell capture in a 1,024 microchamber array using simple gravity flow. No external components are required for assay operation, making our microfluidic approach compatible and easily integrated into the current practices in biological laboratories. High capture rates (from 71% to 84%) were attained for all cell lines tested, making the platform useful as a generic single-cell assay tool. In the platform, cells are individually separated into each chamber; this not only prevents uncontrolled cell-aggregation but also enables tracking of individual cell behaviors within heterogeneous populations.

Although polyHEMA has been used for suspension cell culture for many years, the conventional coating technique (evaporation and dry) provides poor uniformity. Spin-coating and reflow widely used in the field of microfabrication was introduced to control surface uniformity. With the optimized coating protocol, the surface roughness can be reduced down to 0.2 μm, only 6% of the conventional coating process (>3 μm). This high uniformity facilitates robust microfluidic integration.

In the performance of tumorsphere assays, our microfluidic device proves advantages over the conventional approaches on several fronts. First, the hydrodynamic capture scheme simplifies single-cell seeding. Each single cell is captured in its own microchamber, preventing any possible aggregation from skewing sphere formation readout. Second, the presented platform provides a continuous perfusion of media from gravity flow for long-term culture at high-throughput. In conventional approaches, pipetting fresh media or removing waste can potentially disrupt and/or remove cells, complicating or outright preventing media exchange over long culture periods. Our microfluidic approach contains 1,024 microchambers (each chamber is 120 μm by 200 μm) for single-cell sphere formation in an order of magnitude smaller area (15 mm by 12 mm), while 96- or 384-well non-adherent culture plates, which are often used for tumorsphere assays, occupy an area of 127.8 mm by 85.5 mm. Finally, the presented technology greatly improves the reliability and throughput of tumorsphere assays. With this device, we successfully performed single-cell derived sphere assays for A549, C6, HCC38, MCF-7 MDA-MB-231, SKOV3, SUM149, and SUM159 cell lines and 2 patient derived xenograft samples.

We also examined the relationship between Notch expression and single-cell sphere formation. The throughput and reliability facilitated the use of unsorted Notch reporter cells. In this experiment, we observed that Notch+ and Notch- cells both formed spheres, though at significantly different rates, suggesting that there could be progenitor cells forming a portion of the resulting spheres. Subsequent genetic analysis allowed us to identify numerous genes of interest that were different between the two clonal spheres. HES1 and DLL1 are both upstream controllers of Notch signaling through PTEN, so the up-regulation here in the Notch+ derived spheres is consistent with previous literature^45^. Other genes that are upregulated (e.g. YAP1, TM4SF1, TSPAN6, ITGA6, and IL6R) have been correlated with an increase in stemness and tumorigenic potential, suggesting more cells with higher stem-like potential formed Notch+ derived spheres as compared to Notch- derived spheres^47^,^48^,^49^. Up-regulation of CXCR1 in Notch+ derived spheres is also correlated with higher stem-like potential, as it has been shown to be almost exclusively expressed in the CSC population as compared to bulk tumor cells^50^. The overall decrease in CD44 and EpCAM, though, is unexpected. Overexpression of Notch-1 has been shown to contribute to the CSC self-renewal capacity and an acquisition of an EMT-like (epithelial to mesenchymal transition) state^51^. These changes were facilitated by activation of both CD44 and EpCAM^52^, which contradicts the results seen here. It is possible that our sphere culture skews the CSCs within the sphere into a more MET-like (mesenchymal to epithelial transition) state, but larger studies will be required to test this hypothesis. Additionally, media choices (serum versus serum free) and culture conditions (2D adherent versus sphere) greatly affect single cell expression (Supplementary Fig. 13), so future experiments using this approach will require optimization of these parameters.

Due to the single-cell resolution of our data, we are also able to detect a small population of cells expressing ALDH1a3 within the Notch+ derived sphere cells. Enhanced ALDH activity is a hallmark of normal and cancer stem cells, and ALDH1a3 activity, in particular, significantly contributes to the Aldefluor positive phenotype in mouse hematopoietic stem cells and human breast cancer CSC^53^. Within these cells, CXCR1, CXCR4, β-catenin, AKT1, NUMB, and NESTIN, all CSC related genes, are all significantly upregulated as compared to other Notch+ derived cells. It is possible that these cells are progenitor/stem cells (or early descendants) that asymmetrically divided to give rise to the other cells and the heterogeneity observed in the sphere. In addition to the 4 identified ALDH1a3 high Notch+ derived cells, 1 cell was identified to have increased ALDH1a3 expression in the Notch- derived sphere. Though we could not perform ANOVA to determine what genes were significantly different as compared to the other cells in the clone as there was only one cell identified, the ALDH activity hints at greater stem potential, even though it was derived from a Notch- source. Future analysis of such rare cells might provide insight into clonal heterogeneity or possible dissection of elusive de-differentiation events, which has to date proved a great challenge.

While the sample size of our multiplex analysis herein is small, our results and detailed analysis clearly demonstrates the power and utility of our presented approach in the context of CSC biology. Our data also highlights the important applications for our methodology in the investigation of inter- and intra-clonal heterogeneity. Intra-clonal heterogeneity, in particular, has been our focus and is a major challenge in cancer therapy due to the difficulty in studying these phenomena. Spheres formed from our device are clonal in nature, thus allow us to 1) identify potential CSCs through our high throughput tumorsphere assay and then 2) dissociate and analyze the resulting spheres to study the clonal progeny of the CSCs. Our efforts represent a demonstration of the utility of this microfluidic assay; extensive studies of multiple cell lines and intra-/inter-clonal heterogeneity will be explored in the future. Additionally by introducing biological agents of interest or stromal cell conditioned media, our assay can determine how exogenous factors influence CSC sphere formation, clonal evolution, and epigenetic regulation in the progeny. The application of this technology may also prove useful for high throughput screening of therapeutic agents targeting cancer stem cells, using sphere formation as a functional surrogate for CSC inhibition.

## Methods and Materials

### PolyHEMA coating and characterization

Poly(2-hydroxyethyl methacrylate) (polyHEMA) (Sigma-Aldrich, MO, USA) was dissolved at 60 mg/mL in 95% ethanol at 65 °C overnight. 50 μL/cm^2^ was pipetted onto a glass substrate and coated using a 2 step spin process (Solitec spinner): (1) 500 rpm for 10 second (spread), and (2) 1000 rpm for 60 second (spinning). The thickness of polyHEMA layer can be precisely controlled by the spin speed. The spin-coating process was repeated twice to avoid any pinholes on the surface. As the glass transition temperature of polyHEMA is 100 °C, the polyHEMA film was reflowed at 150 °C, well below the burning temperature of polyHEMA (200 °C). For the conventional evaporation coating, procedure 50 μL/cm^2^ of 60 mg/mL polyHEMA solution was pipetted onto a glass substrate and evaporated at room temperature overnight. As polyHEMA is not an ideally reflective material, a 20 nm-thick gold layer was deposited on the polyHEMA-coated substrate to facilitate laser interference characterization. A laser interference microscope (LEXT OLS4000 3D Laser Measuring Microscope, Olympus, Japan) was used to measure the surface profile and surface roughness, which is the arithmetic average of absolute values, using the embedded software. Five measurements on the four corners and the center were performed, and the average and the standard deviation of surface roughness were calculated.

### Device fabrication and assembly

We used standard soft lithography to make PDMS (polydimethylsiloxane) layers and bond them to a polyHEMA coated glass substrate. Two masks were used to fabricate the multiple heights for the channel region (40 μm height) and the capture gap (15 μm height), respectively. The polyHEMA was selectively coated within the culture area via a simple lift-off process using scotch tape to delineate the peripheral region for bonding. The PDMS layer and the polyHEMA-coated glass substrate were bonded together after activation by oxygen plasma treatment (100 Watt, 60 second).

### Cell culture

SKOV3 cells were obtained from Dr. Ronald Buckanovich (University of Michigan, Ann Arbor, MI, USA) and cultured in RPMI with 10% FBS and 1% penicillin/streptomycin. SUM159, SUM149, MDA-MB-231, MCF-7, and T47D-Notch reporter cells were obtained from Dr. Max Wicha (University of Michigan, Ann Arbor, MI, USA). C2C12 cells were obtained from Dr. Ken Pienta (Now at John Hopkins, Baltimore, MD, USA). SUM159, SUM149, MDA-MB-231, MCF7, and C2C12 cells were cultured in DMEM with 10% FBS and 1% penicillin/streptomycin. T47D Notch reporter cells were cultured in RPMI with 10% FBS and 1% penicillin/streptomycin.

### Focal adhesion staining

Millipore’s Actin Cytoskeleton and Focal Adhesion Staining Kit (#FAK100) was used to stain actin (TRITC-conjugated Phalloidin), nucleus (DAPI), and anti-vinculin. MDA-MB-231 cells were cultured to 50–60% confluence, trypsinized, seeded into 60 mm petri-dishes (polyHEMA coated and non-coated), and cultured for 24 hours. For staining, cells were fixed with 4% paraformaldehyde in PBS for 15–20 minutes at room temperature (RT). After washing, cells were permeabilized with 0.1% Triton X-100 in PBS for 5 minutes at RT, washed, and then blocked with 3% BSA for 30 minutes. Cells were stained with anti-vinculin antibody at RT for 1 hour, washed in PBS, and simultaneously incubated with TRITC-conjugated phalloidin and FITC conjugated secondary antibody. After washing, nuclei were stained with DAPI for 1–5 minutes at RT.

### Cell loading process

Cells were harvested from a petri-dish with 0.05% Trypsin/EDTA and centrifuged at 1000 rpm for 5 minutes. Then, cells were re-suspended at 0.5 × 10^6^ cells/mL in culture media, and 100 μL of the cell solution was injected into the inlet of the assay chip. The flow (0.04 μL/min) was generated spontaneously by gravity flow from the liquid height difference (5 mm, 50 Pa pressure) between the inlet and the outlet, so no external pump was required. Within 5 minutes, the cells were hydrodynamically captured in each chamber at single-cell resolution, and the cell solution was replaced with cell-free culture media. To characterize cell capture rate, the cells were stained with 1 μM fluorescent dye (Invitrogen, Cell tracker Green C2925) in the device.

### Single-cell derived sphere experiment

For sphere formation assays, cells were cultured in serum-free MEBM (Lonza, Allendale, NJ) supplemented with B27, 20 ng/ml bFGF, 20 ng/ml EGF, 5 ug/mL insulin, 1 mM lipid concentrate (Invitrogen), 1 ug/mL hydrocortisone, 100 uM mercaptoethanol, 10 uM cholesterol, and 1% penicillin/streptomycin. The cells were monitored for 10 days, and the cell clusters that had grown to at least 50 μm in diameter were scored as a sphere.

### Preparation of patient derived xenograft sample

The University of Michigan has a federally approved Unit for Laboratory Animal Medicine (ULAM) located in the NCRC. Animal housing and care were provided by ULAM. The mouse research have approved animal use protocols in place which have been reviewed by the University Committee for the Use and Care of Animals (UCUCA) as federally regulated. The UCUCA itself is accredited periodically by the AAALAS. All experimental protocols were approved by UCUCA.

Female NOD/SCID mice, 4–8 weeks old, were prepared for surgery the day before injection by using Nair exfoliant to remove hair around the incision site. Mice were anesthetized using isoflurane inhalation with a vaporizer. The incision site was disinfected with betadine and a midline incision was made between the fourth and fifth nipples and then angled toward the nipples. The skin was loosened from the body wall. Patient-derived xenograft (PDX) breast cancer cells (VB, 2147, VARI-068) were suspended in 50 μL of 1:1 DMEM:Matrigel (BD Biosciences) and injected into the inguinal fat pad using a syringe with 18gauge needle. The skin was secured with 9 mm wound clips and clips removed after 7–10 days.

After time for tumor growth, PDX-bearing mice with ~1 cm diameter tumors were euthanized by CO2 asphyxiation. Tumors in the inguinal mammary fat pad were excised, weighed, and <1 g tumor in 60 mm dish was minced with a razor blade to <2 mm pieces. Minced tumor was transferred to a C-tube (Miltenyi Biotec, Auburn, CA) and digested with the Human Tumor Dissociation Kit (Miltenyi Biotec) following the manufacturer instructions. Briefly, each C-tube contained 4.7 mL RPMI-1640 media, 200 μL reagent H, 100 μL reagent R, and 25 μL reagent A. Tumors were digested on the gentle MACS Octo Dissociator with heating elements for 1 hr using the medium tumor setting. Following digestion, 10 mL of RPMI-1640 was added to each C-tube, filtered through a 70 μm strainer (Corning) into a 50 mL conical, and the C-tube rinsed with 10 mL RPMI-1640. Samples were centrifuged at 337 g for 10 min at 4 °C. Pellets were suspended in 10 mL RPMI-1640, filtered through a 40 μm strainer (Corning), and combined with 10 mL rinse of 50 mL tube. Samples were counted on Luna FL (Logos Biosystems, Annandale, VA) using AO/PI and manufacturer instructions. Samples were depleted of mouse cells using the Mouse Cell Depletion Kit (Miltenyi Biotec). Briefly, 10 × 10^6^ cells were suspended in PBS/0.5% BSA, centrifuged at 337 g for 5 min at 4 C, and suspended in 80 μL PBS/0.5% BSA. 20 μL of the mouse cell depletion cocktail was added and incubated 15 min in the refrigerator. While incubating, a LS column (Miltenyi Biotec) was equilibrated on a MultiMACS Cell24 Separator Plus (Miltenyi Biotec) with 3 × 1000 μL PBS/0.5% BSA. Following 15 min incubation, 400 μL PBS/0.5% BSA was added to each sample. The 500 μL sample was applied to the LS column and the flow through collected in 5 mL FACS tube (Corning). LS columns were washed with 3 × 1000 μL PBS/0.5% BSA and collected into same 5 mL FACS tube. Samples were centrifuged at 337 g for 5 min at 4 C, suspended in 5–10 mL PBS/0.5% BSA, and counted on Luna FL using AO/PI. These methods produce healthier mouse cell free PDX samples

### Lentiviral transduction

T47D cells were transduced with pGreenFire1-Notch lentiviral (System Biosciences, Mountain View, CA) particles using standard protocols. Lentiviruses were prepared using 3rd generation helper plasmids to generate VSVG pseudotyped particles (roughly 1 × 10^7^ units/mL) by the University of Michigan Vector Core. 500,000 T47D breast cancer cells/well (50,000/cm^2^) were plated in a 6-well plate, transduced the following day at a MOI of 10 for 24 hr. Transduction efficiency was ~90% at 1 week post transduction based on FACS analysis of eGFP from cells transduced with pGreenFire-CMV. GFP+ cells were collected by flow cytometry sorting using a MoFlo Astrios cytometer to insure all cells contain the lentiviral vector. Following cell culture, GFP- cells were generated from GFP+ cells after reaching equilibrium.

### Sphere retrieval, dissociation

After 2 weeks culture, a percentage of spheres formed from single cells. As the substrate-PDMS bonding strength is weak in the central polyHEMA coated region (compared to the PDMS-glass bonding) (Supplementary Fig. 5a), the PDMS could be detached by cutting the PDMS on the peripheral bonded region. The spheres were retrieved and placed in a polyHEMA coated non-adherent 384-well plate. The single-cell derived spheres were further grown in the non-adherent well for additional 2 weeks. This provided a sufficient cell number for single cell gene expression analysis using the Fluidigm C1 and Biomarker machine. Spheres containing 200–500 cells were dissociated into single cells using both mechanical pipetting and a 5 minute trypsinization.

### Single cell gene expression analysis

Spheroids derived single cells were loaded onto the C1 chip and processed by the C1 instrument to isolate single cells. All the chambers of C1 chip were examined under the IX83 fluorescent microscope to record the status of captured cells in each chamber. Single cells underwent lysis, RNA release, reverse transcription, and finally cDNA pre-amplification for 96 target gene transcripts in the C1 chip. The pre-amplified cDNAs from each single cell were analyzed by the BioMark HD instrument, which generates nearly 10,000 qPCR data-points in a single run using a 96 × 96 chip and TaqMan assays. Technical and biological replicates shown in Supplement Fig. 14 have been performed to ensure reproducible single cell transcriptome analysis. Serial dilution experiments of total RNA extracted from T47D breast cancer cell line using BioMark HD system and TaqMan assays have been performed to confirm the experiment is quantitative and sensitive (Supplement Fig. 15–18).

The 96-gene panel was selected based on relevant literature findings and optimized to minimize conflict for the available TaqMan assays using computational analysis from Life Technologies^54^. If the results showed conflict between assays, we identified another available assay for the same gene(s) and submitted the panel again for analysis, or we exclude the gene(s) that show conflict and replaced with other target gene(s) with no conflict. The final panel has been tested with T47D (analyzed here), SUM159 (basal claudin low), SUM149 (basal), BT474 (HER2 over expressed) and MCF7 (luminal) cells, resulting in a distinct gene expression signature correspond to each subtype that has been consistent across numerous distinct experiments. TaqMan probes, which are highly specific, were utilized over SYBR Green or Eva Green dye-based PCR methodologies to further limit the potential for non-specific amplicons and prevent the need for post-PCR melting curve assays or gel-electrophoresis confirmation. Gene expression data were analyzed using SINGuLAR and R script to generate PCA, heatmap and violin plots based on the log2 expression value for each gene per single cell.

### Simulations of cell capture scheme

Flow velocity and pressure were simulated to evaluate cell capture scheme in microchambers by COMSOL 4.3. The boundary condition of the inlet was 50 Pa pressure, and that of outlet was 0 Pa. All the sidewalls were set to wall in simulation. The chamber structure was automatically meshed by COMSOL and the simulated in the stationary mode.

### Statistical analysis

Statistical analyses were performed using R (version 3.0). One-way ANOVA tests were used for all comparisons and significance level of p < 0.05 was used to consider statistical significance. Results are presented as mean ± SD. Measurements with high variability (such as gene expression levels) were compared on the log-scale. For single-cell qRT-PCR data generated from the Fluidigm Biomark HD system, we used SINGuLAR v3.0 for exploratory data analysis, such as outlier detection, hierarchical clustering and principal component analysis. R package SingleCellAssay was used for its improved statistical power in detecting differentially expressed genes.

## Additional Information

**How to cite this article**: Chen, Y.-C. *et al*. High-Throughput Single-Cell Derived Sphere Formation for Cancer Stem-Like Cell Identification and Analysis. *Sci. Rep.* **6**, 27301; doi: 10.1038/srep27301 (2016).

## Supplementary Material

Supplementary Movie 1

Supplementary Movie 2

Supplementary Information

## Figures and Tables

**Table 1:** Single cell-derived sphere formation rates of breast cancer cell lines and breast cancer derived PDX samples.

Cell Lines	Sphere Formation Rate (%)	PDXs	Sphere Formation Rate (%)
SUM159	55.4 ± 6.8	PDX# 2147	1.8 ± 1.3
MCF-7	3.1 ± 2.5		
T47D	2.8 ± 1.1	PDX# VARI068	0.8 ± 0.6
SUM149	1.8 ± 0.8		
HCC38	1.2 ± 0.8		
MDA-231	1.4 ± 0.6		
