# Supplementary material for: High-Throughput Single-Cell Derived Sphere Formation for Cancer Stem-Like Cell Identification and Analysis
Source: Sci Rep. 2016 Jun 13;6:27301. doi: 10.1038/srep27301 (PMC4904376; doi:10.1038/srep27301)
Supplement: Supplementary Information [file srep27301-s3.pdf]

High-Throughput Single-Cell Derived Sphere Formation for Cancer Stem-Like Cell Identification and Analysis

Yu-Chih Chen[1,3*], Patrick N. Ingram[2*], Shamileh Fouladdel[3], Sean P. McDermott[3], Ebrahim Azizi[3], Max S. Wicha[3] and Euisik Yoon[1,2]
[1]Department of Electrical Engineering and Computer Engineering, University of Michigan, 1301 Beal Avenue, Ann Arbor, MI 48109-2122;
[2]Dept. of Biomedical Engineering, University of Michigan, Ann Arbor, MI, USA, 2200 Bonisteel, Blvd. Ann Arbor, MI 48109-2099
[3]University of Michigan Comprehensive Cancer Center, 1500 East Medical Center Drive, Ann Arbor, MI 48109-5940
[*]Equal contribution as first authors

Corresponding author:
Euisik Yoon, 1301 Beal Avenue, Ann Arbor, MI 48109-2122, 734-615-4469, esyoon@umich.edu.

Keywords:
Microfluidics, Cancer Stem Cells, Suspension Culture, Sphere Culture, Single Cell, Surface Modification, PolyHEMA

SUPPLEMENTARY INFORMATION

Cell Lines	Cell Capture Rate (%)
SUM159	74.1±8.6
MDA-231	83.2±11.3
MCF-7	67.8±18.5
SKOV3	81.7±7.5
C2C12	70.1±3.2

Supplementary Table 1: Single cell capture rates of 5 different cell lines utilizing passive hydrodynamic capture in the single cell devices. Data are shown as the mean ± SD (N = 4).

	Evaporation Process	Proposed Process
Thickness	30.5±2.71 μm	2.04±0.26 μm
Roughness RMS***	3.15±0.50 μm	0.19±0.04 μm
Peak Height	6.55±0.62 μm	0.56±0.07 μm
Valley Depth	9.24±1.65 μm	0.65±0.14 μm

Supplementary Table 2: The comparison between the surface roughness of conventional polyHEMA coating techniques and the presented improved methodology. Data are shown as the mean ± SD (N = 5), *** $P < 0.001$.

Cell Lines	Sphere Formation Rate (%)
A549	0.6±0.3
SKOV3	2.3±2.1
C6	2.6±2.1

Supplementary Table 3: Single cell-derived sphere formation rates of non-breast cancer cell lines. A549 is lung cancer cell line; SKOV3 is ovarian cancer cell line; and C6 is brain tumor cell line.

Gene	P-Value
**EpCAM**	1.9E-23
**STAP2**	9.5E-17
**TAZ**	3.9E-12
**CD14**	7.2E-09
**ID1**	8.8E-09
**YAP1**	4.1E-07
**CCND1**	7.8E-06
**TM4SF1**	8.0E-06
**TSPAN6**	1.5E-05
**ITGA6**	9.1E-05
**IL6R**	1.3E-04
**ID2**	2.6E-04
**CD24**	7.5E-04
**CXCR1**	3.2E-03
**CDH1**	3.7E-03
**TNKS1BP1**	7.5E-03
**CD44**	1.4E-02
**GAPDH**	1.6E-02
**PTEN**	3.0E-02
**HES1**	3.6E-02

Supplementary Table 4: The p-values of genes that are significantly different between Notch+ sphere derived cells and Notch- sphere derived cells for Figure 5.

Gene	P-Value
**ALDH1a3**	3.4E-12
**bCatenin**	1.7E-04
**AKT1**	8.1E-04
**CXCR1**	8.3E-04
**CXCR4**	1.5E-03
**TCEA3**	3.0E-03
**GAPDH**	3.5E-03
**PPL**	5.7E-03
**p53**	1.0E-02
**NUMB**	1.1E-02
**DLL3**	1.6E-02
**NESTIN**	1.6E-02
**TGFb**	1.6E-02
**DLL4**	1.6E-02
**TNKS1BP1**	2.0E-02
**Notch1**	2.3E-02
**TAZ**	2.5E-02
**EGFR**	2.6E-02
**STAP2**	3.2E-02
**JAG2**	5.2E-02
**NYNRIN**	5.6E-02
**TM4SF1**	5.7E-02

Supplementary Table 5: The p-values of genes that are significantly different in Notch+/ALDH1a3+ cells as compared to Notch+/ALDH1a3- cells in Figure 5c,d.

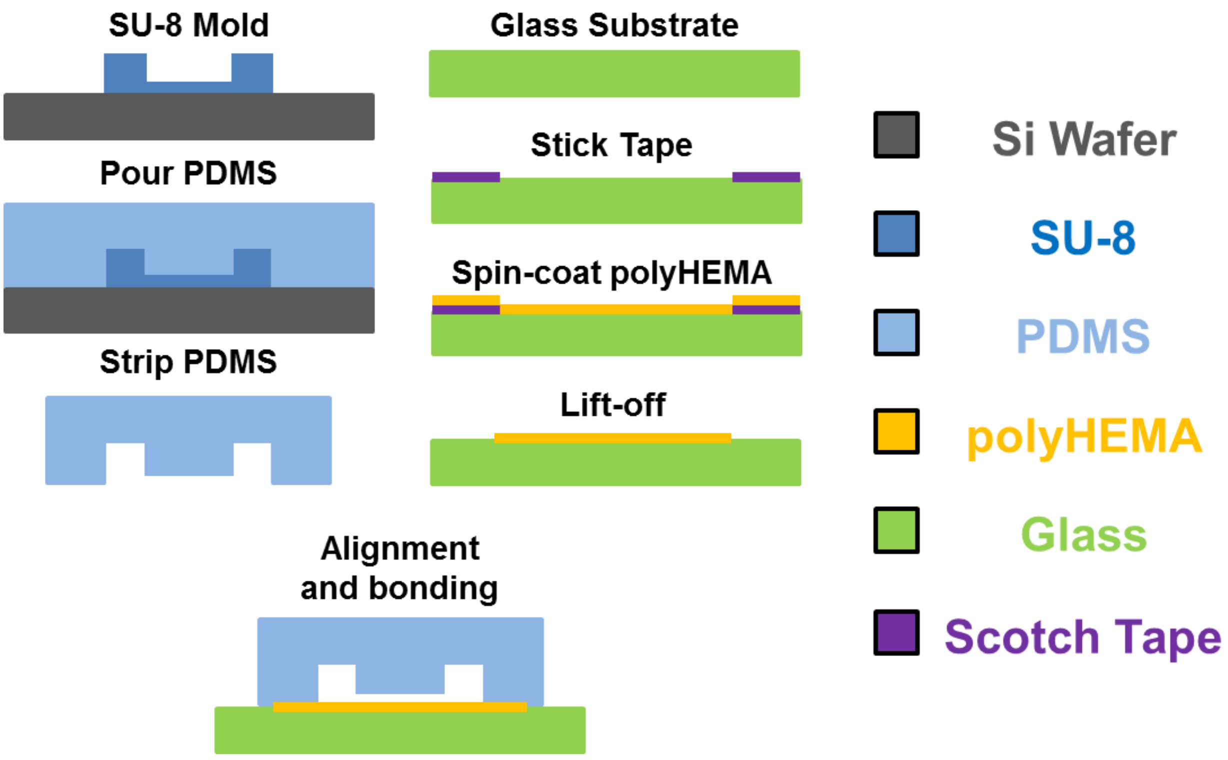

Supplementary Figure 1: Fabrication process of high throughput single cell suspension culture device for mammosphere assays. SU-8 mold is fabricated in a 2-step photolithographic process. PDMS is poured over the mold and cured. After overnight curing, the PDMS is removed from the mold for bonding to the substrate. The glass substrate (microscope slide) is cleaned and masked with scotch tape to restrict the polyHEMA pattern to the area of the microchambers. A polyHEMA ethanol solution (60mg/mL) is spin-coated (1000 rpm for 60 second) over the substrate and reflowed at 150°C after the tape mask is removed. After oxygen plasma oxygenation (100 Watt, 60 second), the substrate and PDMS layer are bonded.

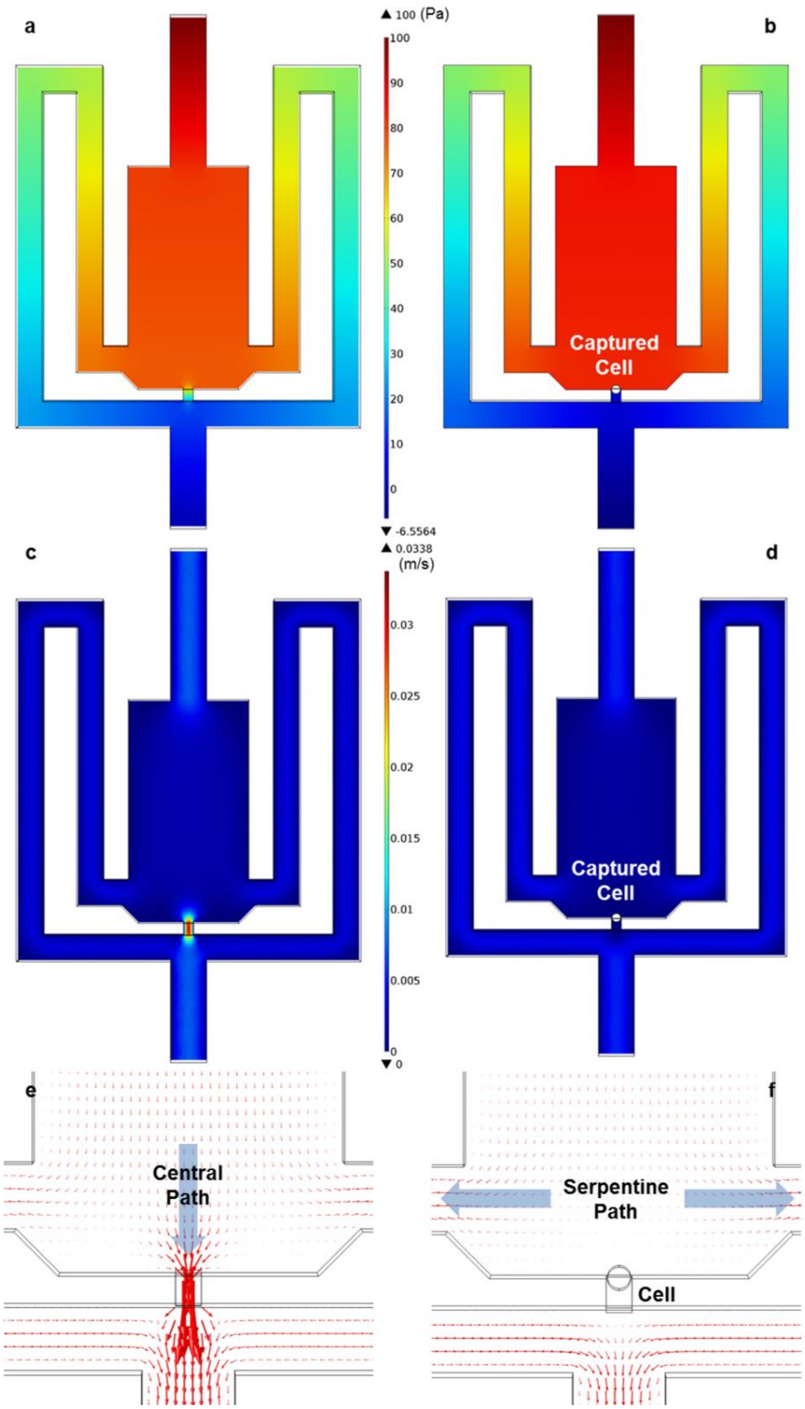

Supplementary Figure 2: Simulations of flow velocity and pressure to evaluate cell capture scheme. (a, b) Simulations of pressure distribution before and after cell capture. (c, d) Simulations of flow velocity before and after cell capture. (e) The simulated flow pattern before cell capture. The red arrows indicating flow direction and velocity suggest that the cells are likely to be guided to the capture site and get captured. (f) After cell capture, the captured cell blocks the flow, so the next cell will be guided into the serpentine path.

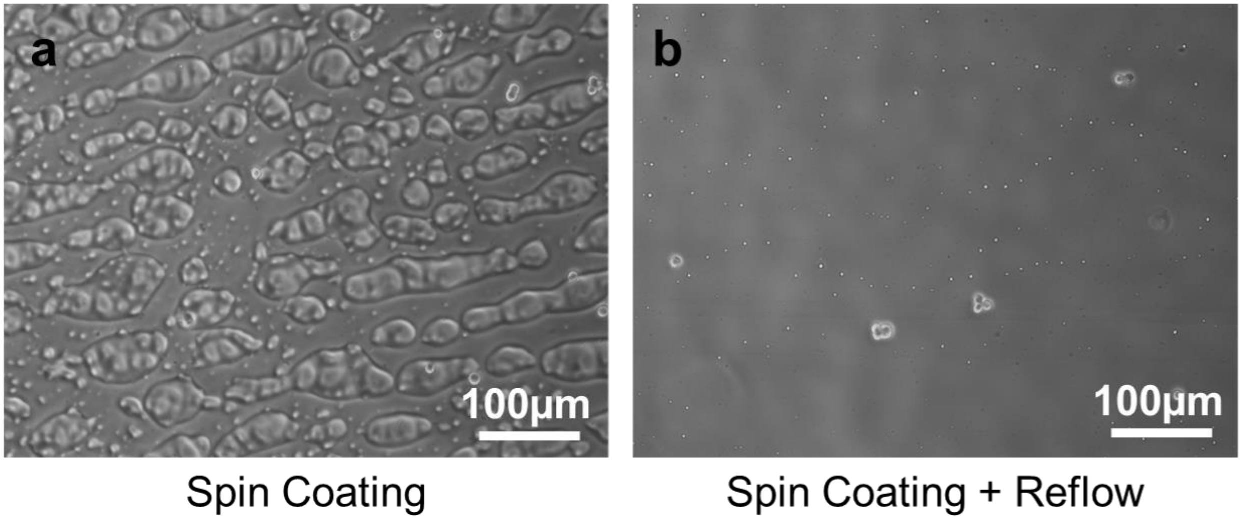

Supplementary Figure 3: PolyHEMA spin-coated surface with and without reflow process: (a) the polyHEMA spin-coated surface without reflow. As the ethanol evaporates fast during the spin-coating, the radial trenches are formed on the substrate. Also, some evaporation bubbles were trapped in the polyHEMA film, making the surface coarse. (b) After reflow at 200 °C overnight, the trench profile was flattened, and the trapped bubbles were released, making the coated surface smoother.

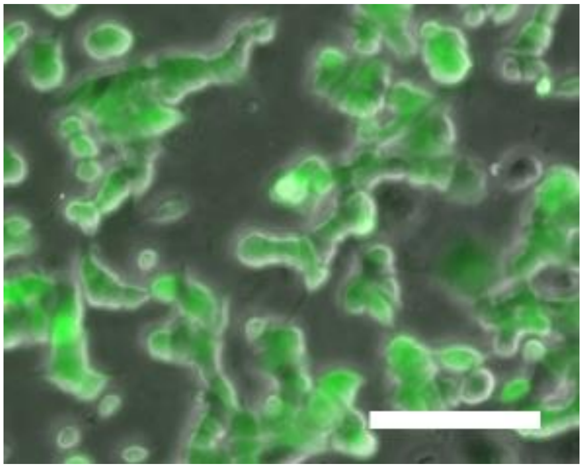

Supplementary Figure 4. T47D culture on blank PDMS (scale bar: 100 μm)

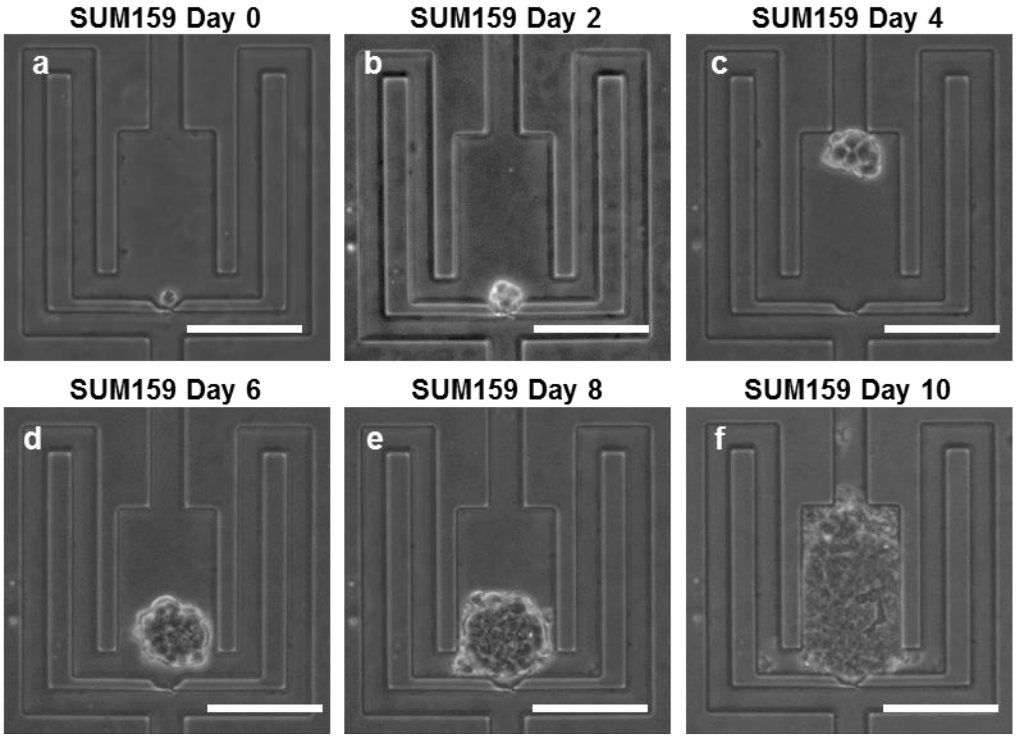

Supplementary Figure 5: Formation and morphology of cancer spheres derived from a single SUM159 breast cancer cell: (a) day 0, (b) day 2, (c) day 4, (d) day 6, (e) day 8, and (f) day 10.

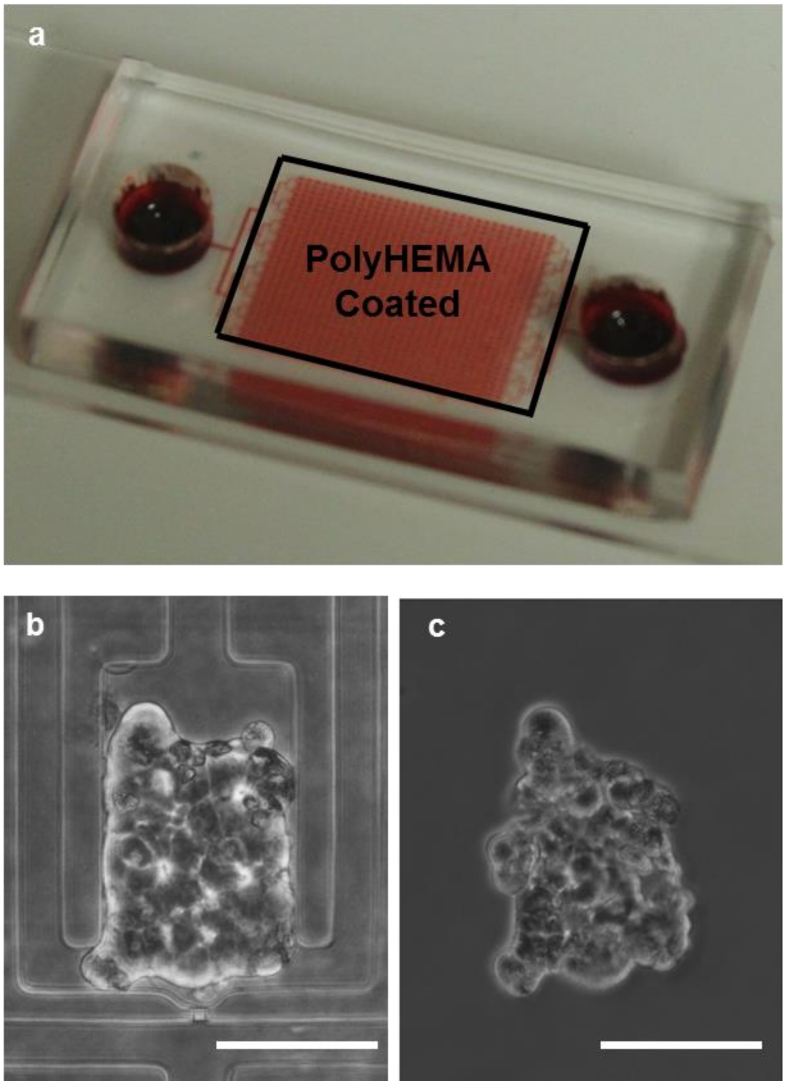

Supplementary Figure 6: Single cell derived sphere retrieval process: (a) Photograph of fabricated 1,024 well suspension culture device with polyHEMA coated area shown. (b) Example of single cell derived sphere in microchamber. After 14 days of culture, the micro-chamber might restrict further sphere growth. (c) Example sphere of interest in multiwall plate after release and retrieval from microfluidic platform.

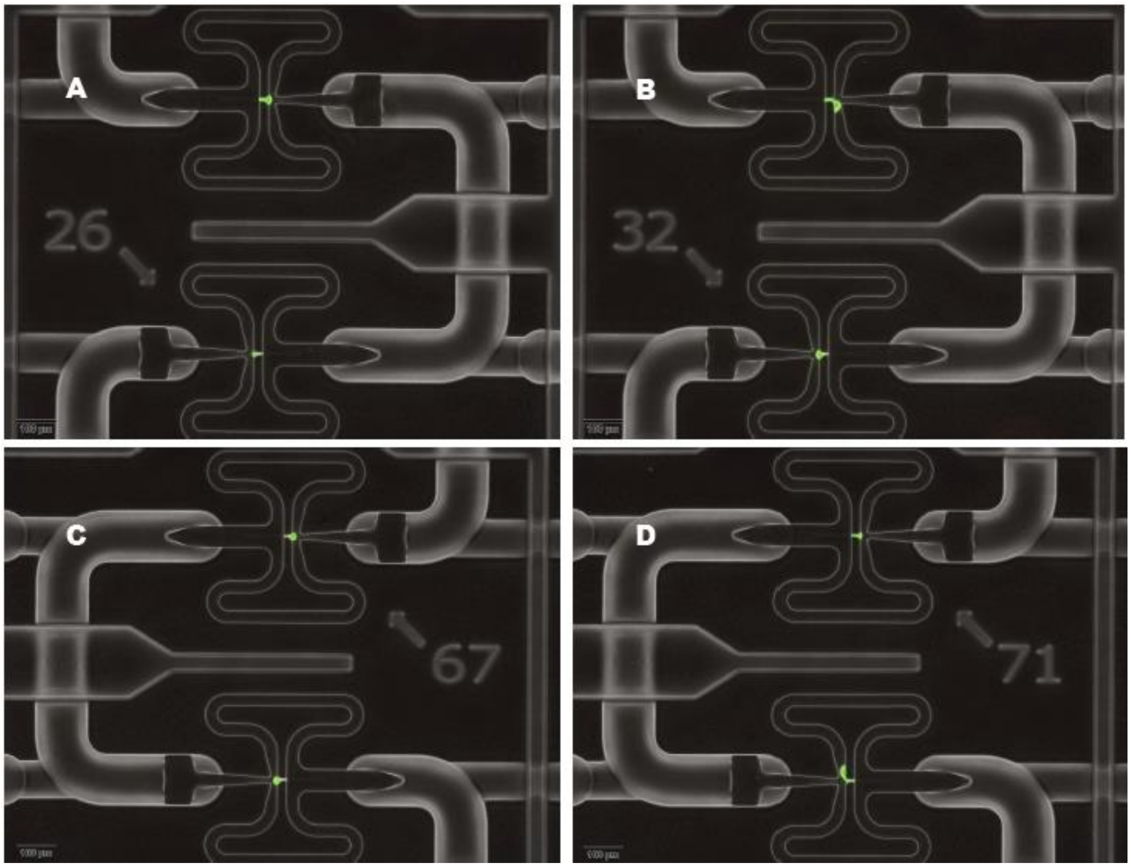

Supplementary Figure 7. Representative images of single breast cancer cells isolated within the C1 chip. (a) Large versus small size round shape single cells, (b) elongated versus round shape single cells, (c) similar size round shape single cells (d) and small round shape versus large elongated single cells.

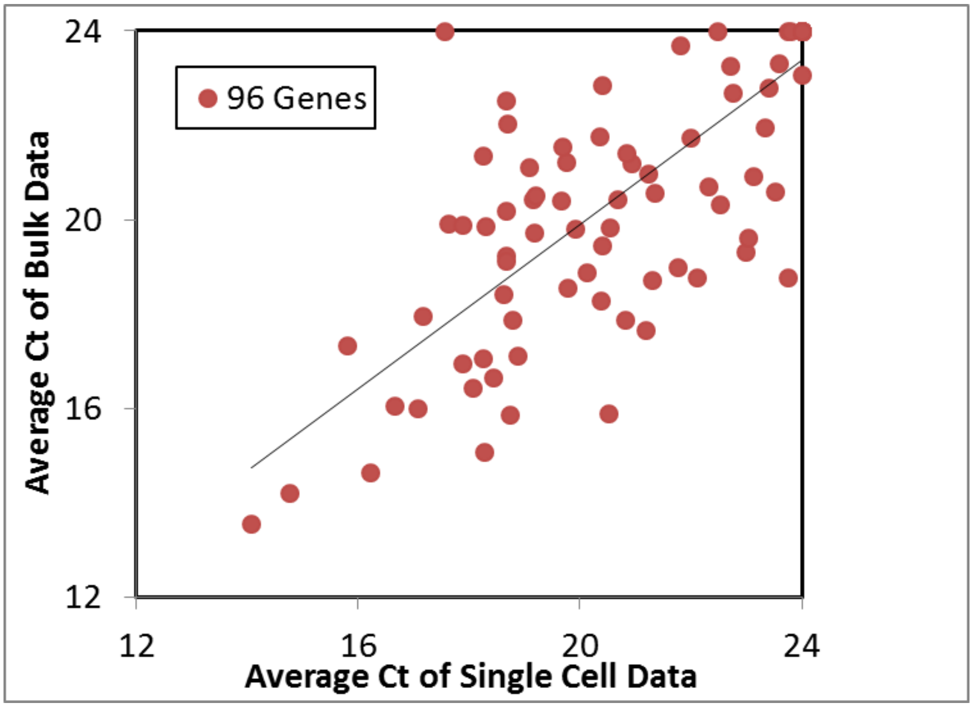

Supplementary Figure 8. Correlation between single T47D cell (n = 28) expression data and bulk T47D (5,000 cells each sample, n = 3) expression data (both normalized to the geometric mean of GAPDH and RAB7A). The average Ct of 96 genes are highly correlated (correlation coefficient r = 0.79) between single cell and bulk data.

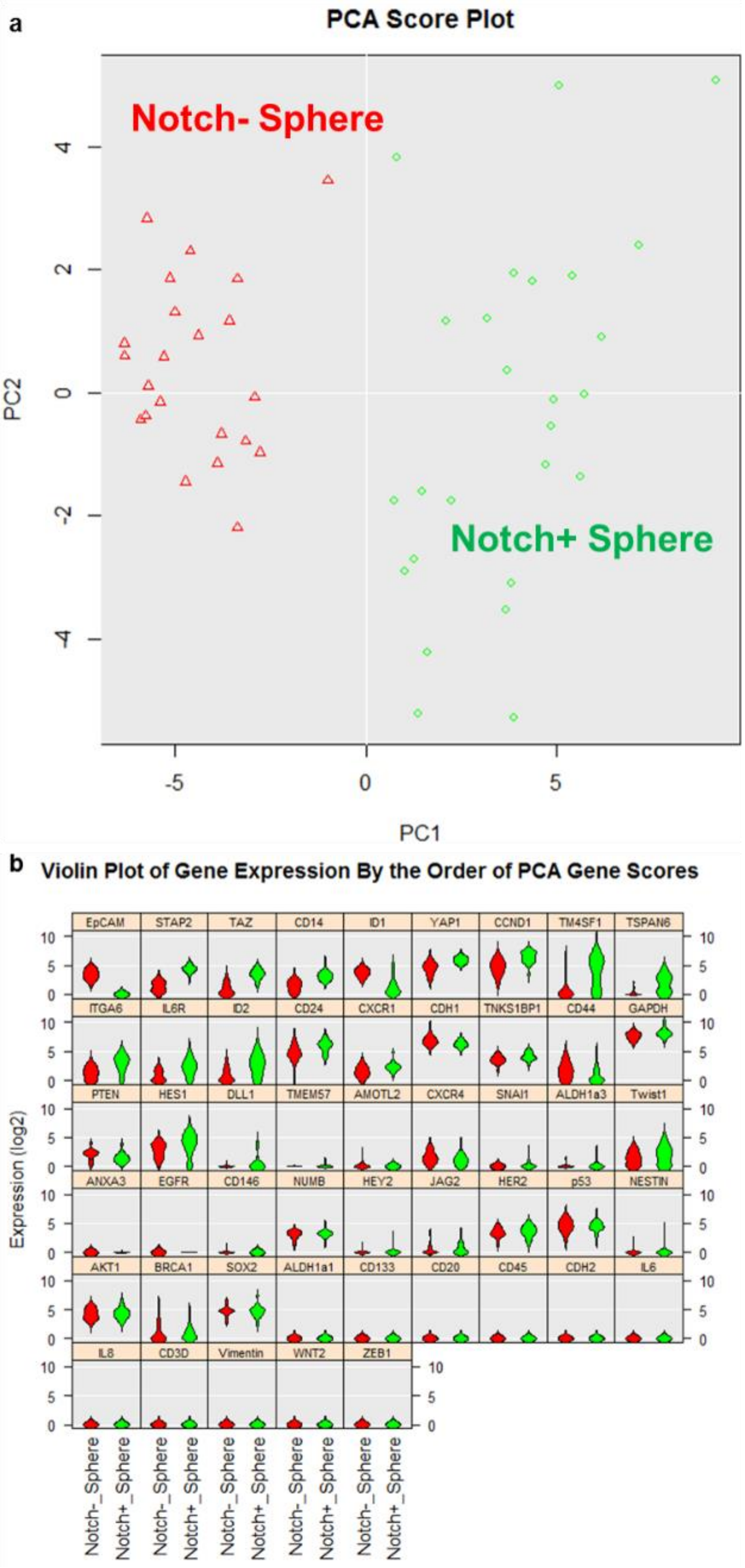

a
PCA Score Plot
Notch- Sphere
Notch+ Sphere
PC2
PC1
4
2
0
-2
-4
-5
0
5
b
Violin Plot of Gene Expression By the Order of PCA Gene Scores
Expression (log2)
EpCAM STAP2 TAZ CD14 ID1 YAP1 CCND1 TM4SF1 TSPAN6
ITGA6 IL6R ID2 CD24 CXCR1 CDH1 TNKS1BP1 CD44 GAPDH
PTEN HES1 DLL1 TMEM57 AMOTL2 CXCR4 SNAI1 ALDH1a3 Twist1
ANXA3 EGFR CD146 NUMB HEY2 JAG2 HER2 p53 NESTIN
AKT1 BRCA1 SOX2 ALDH1a1 CD133 CD20 CD45 CDH2 IL6
IL8 CD3D Vimentin WNT2 ZEB1
10
5
0
Notch-_Sphere
Notch+_Sphere

Supplementary Figure 9: Expression data of single cells from retrieved spheres utilizing Fluidigm C1/Biomark HD for multiplexed gene expression analysis comparing cells derived from T47D Notch+ cells and those derived from T47D Notch- cells. (a) PCA clustering of single cell expression analysis for Notch+ cells compared to cells derived from Notch- cells. (b) Violin plots of 50 genes for differences between Notch+ derived sphere cells and Notch- derived sphere cells.

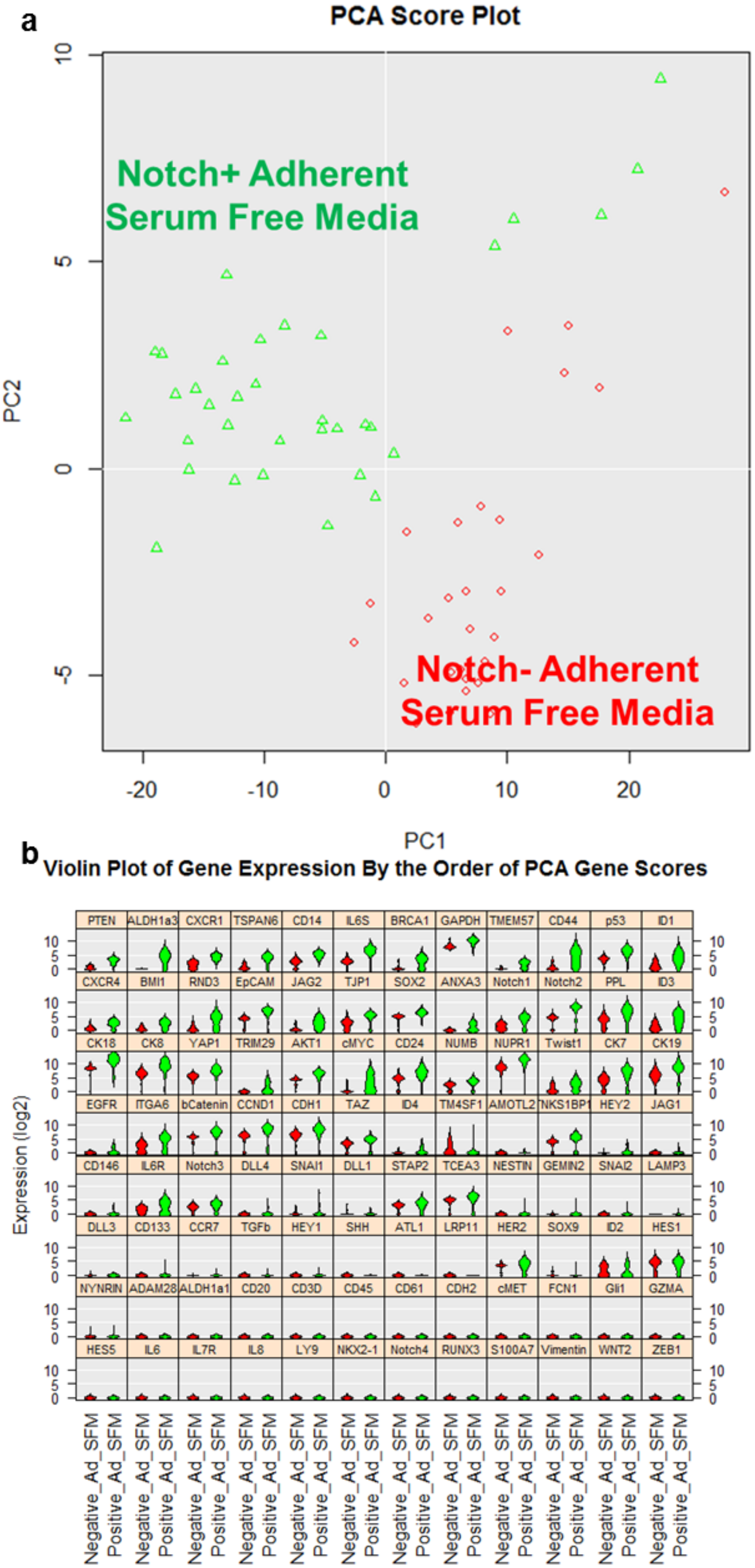
a
PCA Score Plot
Notch+ Adherent
Serum Free Media
Notch- Adherent
Serum Free Media
PC1
PC2
b
Violin Plot of Gene Expression By the Order of PCA Gene Scores
PTEN ALDH1a3 CXCR1 TSPAN6 CD14 IL6S BRCA1 GAPDH TMEM57 CD44 p53 ID1
CXCR4 BMI1 RND3 EpCAM JAG2 TJP1 SOX2 ANXA3 Notch1 Notch2 PPL ID3
CK18 CK8 YAP1 TRIM29 AKT1 cMYC CD24 NUMB NUPR1 Twist1 CK7 CK19
EGFR ITGA6 bCatenin CCND1 CDH1 TAZ ID4 TM4SF1 AMOTL2 NKS1BP1 HEY2 JAG1
CD146 IL6R Notch3 DLL4 SNAI1 DLL1 STAP2 TCEA3 NESTIN GEMIN2 SNAI2 LAMP3
DLL3 CD133 CCR7 TGFb HEY1 SHH ATL1 LRP11 HER2 SOX9 ID2 HES1
NYNRIN ADAM28 ALDH1a1 CD20 CD3D CD45 CD61 CDH2 cMET FCN1 Gli1 GZMA
HES5 IL6 IL7R IL8 LY9 NKX2-1 Notch4 RUNX3 S100A7 Vimentin WNT2 ZEB1
Expression (log2)
Negative_Ad_SFM
Positive_Ad_SFM

Supplementary Figure 10: Single cell expression data from Notch+ and Notch- cells in adherent culture in serum-free media utilizing Fluidigm C1/Biomark HD for multiplexed gene expression analysis. (a) PCA clustering of single cell expression analysis for Notch+ and Notch- adherent cells. (b) Violin plots of the entire 96-gene panel for differences between Notch+ and Notch- cells from adherent culture.

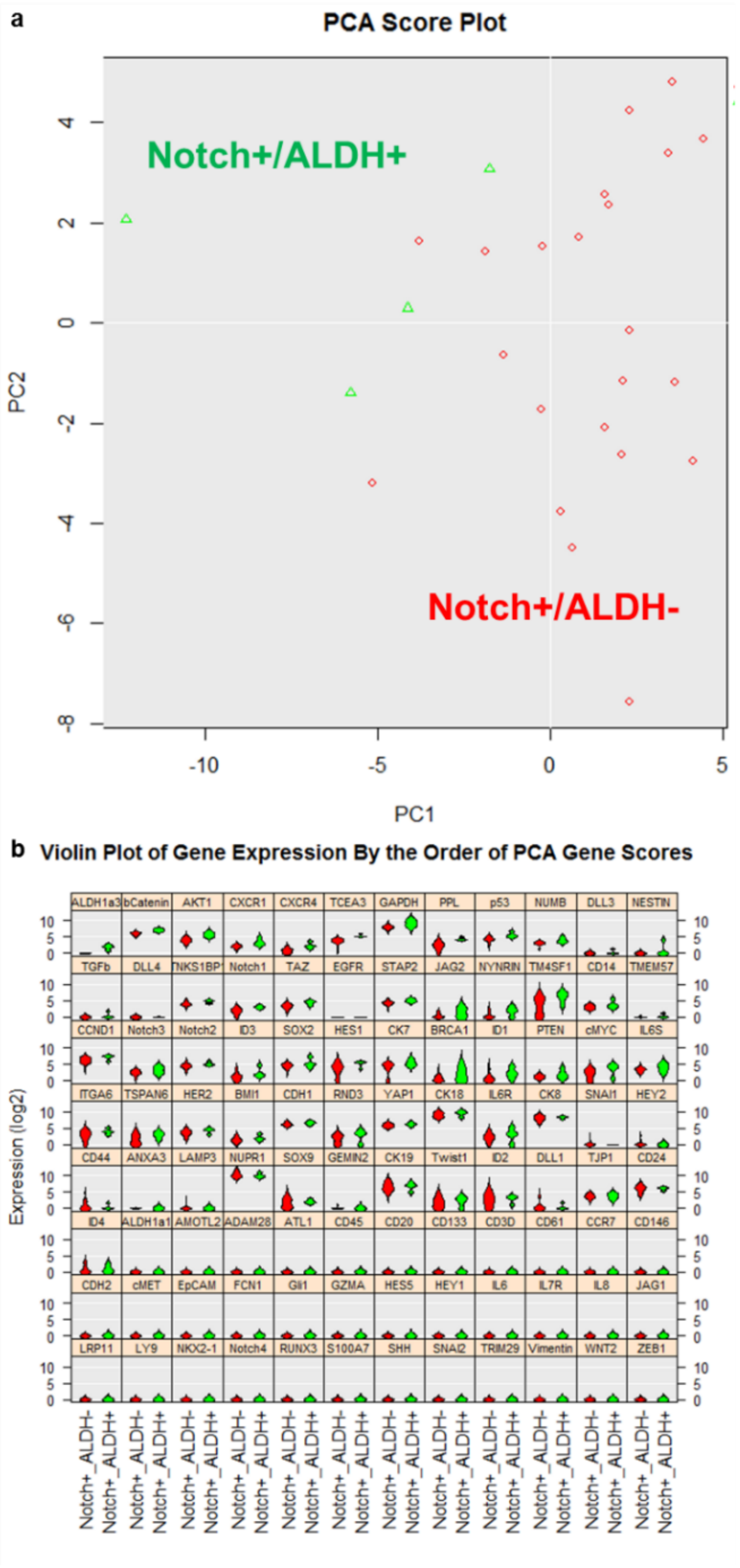
a
PCA Score Plot
Notch+/ALDH+
Notch+/ALDH-
PC1
PC2
b
Violin Plot of Gene Expression By the Order of PCA Gene Scores
Expression (log2)
ALDH1a3 bCatenin AKT1 CXCR1 CXCR4 TCEA3 GAPDH PPL p53 NUMB DLL3 NESTIN
TGFb DLL4 NKS1BP Notch1 TAZ EGFR STAP2 JAG2 NYNRIN TM4SF1 CD14 TMEM57
CCND1 Notch3 Notch2 ID3 SOX2 HES1 CK7 BRCA1 ID1 PTEN cMYC IL6S
ITGA6 TSPAN6 HER2 BMI1 CDH1 RND3 YAP1 CK18 IL6R CK8 SNAI1 HEY2
CD44 ANXA3 LAMP3 NUPR1 SOX9 GEMIN2 CK19 Twist1 ID2 DLL1 TJP1 CD24
ID4 ALDH1a1 AMOTL2 ADAM28 ATL1 CD45 CD20 CD133 CD3D CD61 CCR7 CD146
CDH2 cMET EpCAM FCN1 Gli1 GZMA HES5 HEY1 IL6 IL7R IL8 JAG1
LRP11 LY9 NKX2-1 Notch4 RUNX3 S100A7 SHH SNAI2 TRIM29 Vimentin WNT2 ZEB1
Notch+_ALDH-
Notch+_ALDH+

Supplementary Figure 11: Single cell expression data of retrieved spheres utilizing Fluidigm C1/Biomark HD for multiplexed gene expression analysis comparing Notch+ALDH1a3 high (N=4) and Notch+ALDH1a3 low (N=22) cells. (a) PCA clustering of single cell expression analysis for Notch+ derived ALDH high cells versus ALDH low cells. (b) Violin plots of the entire 96 gene panel for differences between Notch+ derived ALDH high cells versus ALDH low cells

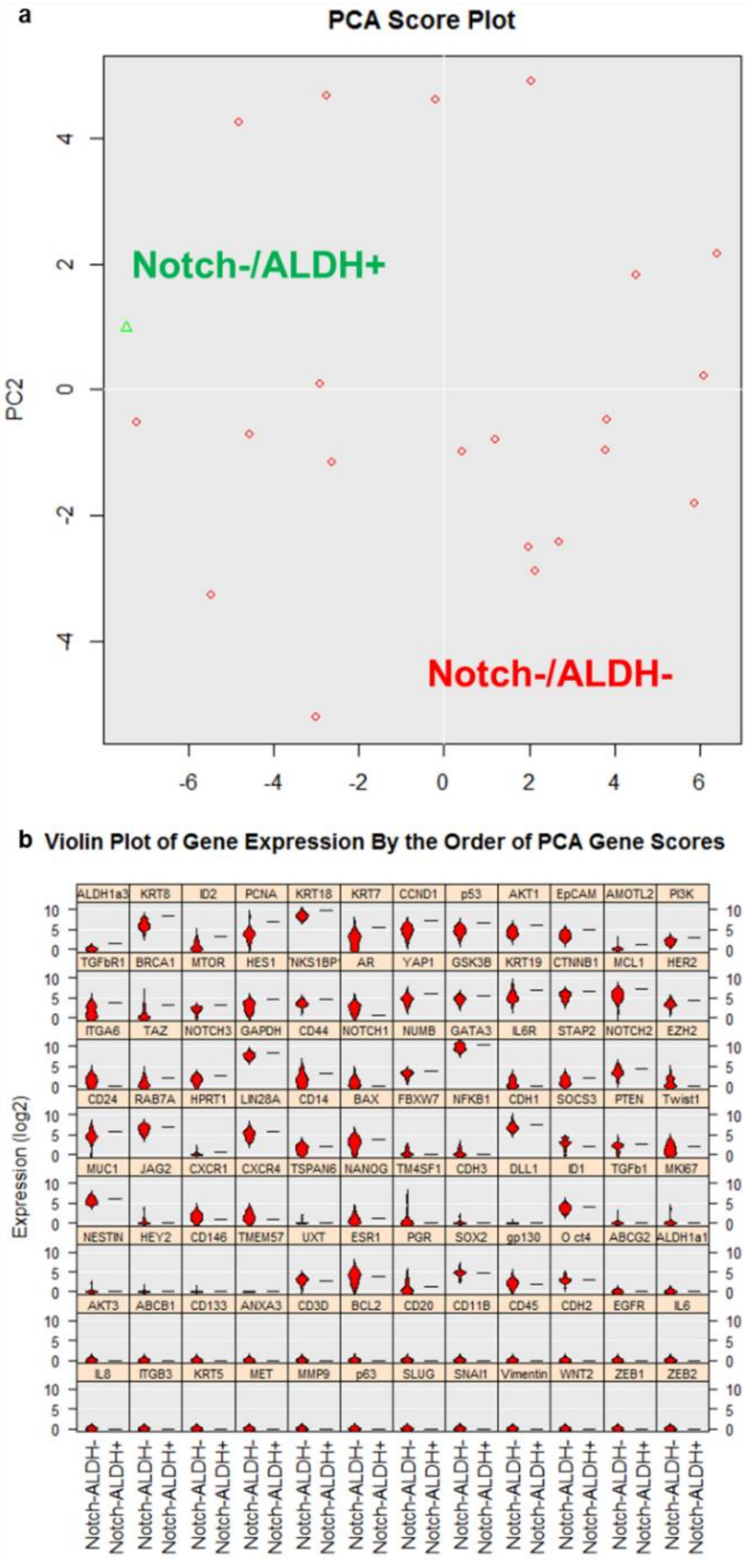
a
PCA Score Plot
Notch-/ALDH+
Notch-/ALDH-
PC2
b Violin Plot of Gene Expression By the Order of PCA Gene Scores
Expression (log2)
ALDH1a3 KRT8 ID2 PCNA KRT18 KRT7 CCND1 p53 AKT1 EpCAM AMOTL2 PI3K
TGFbR1 BRCA1 MTOR HES1 NKS1BP AR YAP1 GSK3B KRT19 CTNNB1 MCL1 HER2
ITGA6 TAZ NOTCH3 GAPDH CD44 NOTCH1 NUMB GATA3 IL6R STAP2 NOTCH2 EZH2
CD24 RAB7A HPRT1 LIN28A CD14 BAX FBXW7 NFKB1 CDH1 SOCS3 PTEN Twist1
MUC1 JAG2 CXCR1 CXCR4 TSPAN6 NANOG TM4SF1 CDH3 DLL1 ID1 TGFb1 MKI67
NESTIN HEY2 CD146 TMEM57 UXT ESR1 PGR SOX2 gp130 O ct4 ABCG2 ALDH1a1
AKT3 ABCB1 CD133 ANXA3 CD3D BCL2 CD20 CD11B CD45 CDH2 EGFR IL6
IL8 ITGB3 KRT5 MET MMP9 p63 SLUG SNAI1 Vimentin WNT2 ZEB1 ZEB2
Notch-ALDH-
Notch-ALDH+

Supplementary Figure 12: Single cell expression data of Notch-ALDH1a3 high and Notch-/ALDH1a3 low cells from Notch- retrieved sphere utilizing Fluidigm C1/Biomark HD for multiplexed gene expression analysis. (a) PCA clustering of single cell expression analysis for Notch-ALDH1a3 high (N=1) and Notch-/ALDH1a3 low (N=21) cells. (b) Violin plots of the entire 96-gene panel for Notch-/ALDH1a3 high cell versus Notch-/ALDH1a3 low cells. Due to only one Notch-ALDH1a3 high cell, there was no statistical comparison possible.

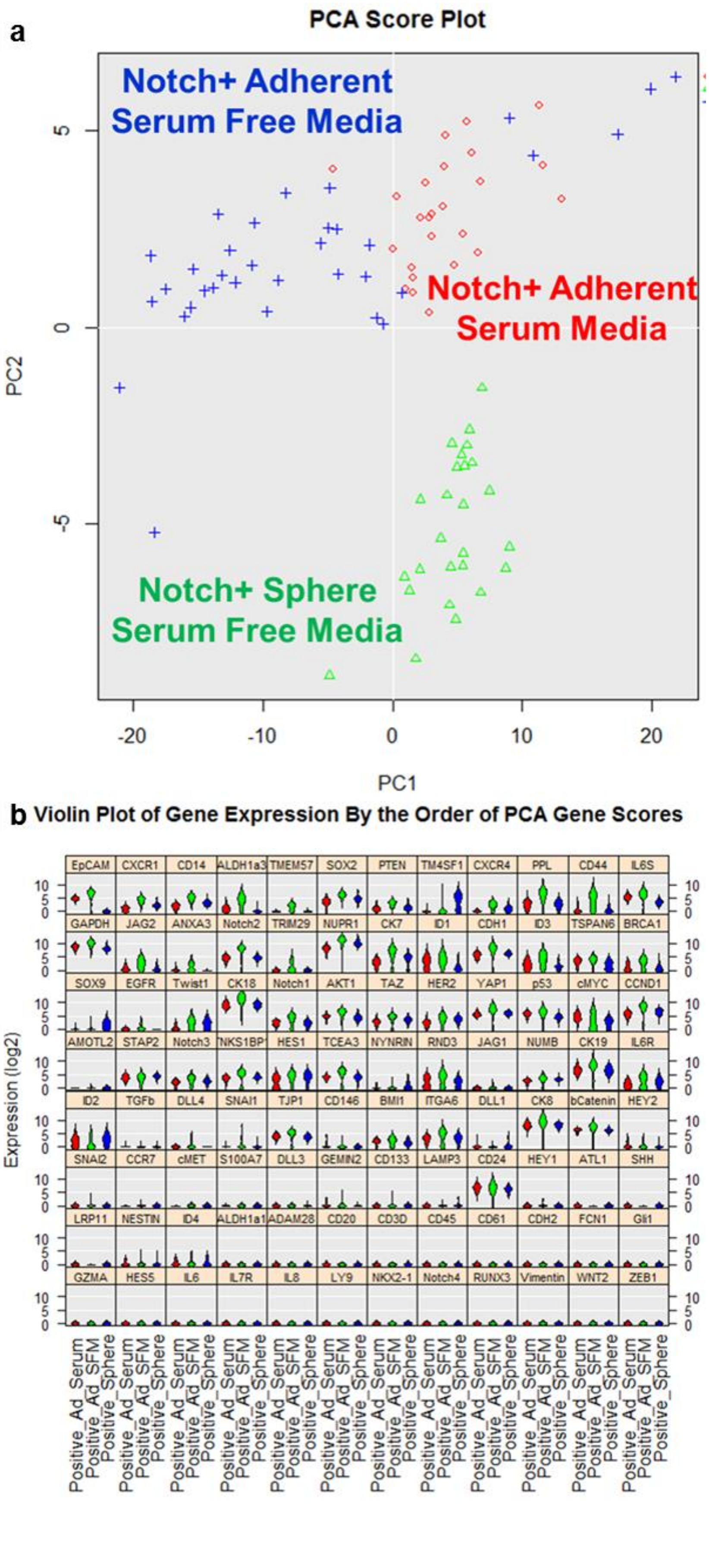
a
PCA Score Plot
Notch+ Adherent
Serum Free Media
Notch+ Adherent
Serum Media
Notch+ Sphere
Serum Free Media
PC2
PC1
5
0
-5
-20
-10
0
10
20
b Violin Plot of Gene Expression By the Order of PCA Gene Scores
Expression (log2)
EpCAM CXCR1 CD14 ALDH1a3 TMEM57 SOX2 PTEN TM4SF1 CXCR4 PPL CD44 IL6S
GAPDH JAG2 ANXA3 Notch2 TRIM29 NUPR1 CK7 ID1 CDH1 ID3 TSPAN6 BRCA1
SOX9 EGFR Twist1 CK18 Notch1 AKT1 TAZ HER2 YAP1 p53 cMYC CCND1
AMOTL2 STAP2 Notch3 NKS1BP HES1 TCEA3 NYNRIN RND3 JAG1 NUMB CK19 IL6R
ID2 TGFb DLL4 SNAI1 TJP1 CD146 BMI1 ITGA6 DLL1 CK8 bCatenin HEY2
SNAI2 CCR7 cMET S100A7 DLL3 GEMIN2 CD133 LAMP3 CD24 HEY1 ATL1 SHH
LRP11 NESTIN ID4 ALDH1a1 ADAM28 CD20 CD3D CD45 CD61 CDH2 FCN1 Gli1
GZMA HES5 IL6 IL7R IL8 LY9 NKX2-1 Notch4 RUNX3 Vimentin WNT2 ZEB1
10
5
0
Positive_Ad_Serum
Positive_Ad_SFM
Positive_Sphere

Supplementary Figure 13: Single cell expression data of T47D Notch+ cells from spheres and adherent culture comparing serum and serum free media conditions utilizing Fluidigm C1/Biomark HD for multiplexed gene expression analysis. (a) PCA clustering of single cell expression analysis for Notch+ derived cells in adherent culture with and without serum in the media compared to Notch+ derived cells from spheres in serum free media. (b) Violin plots of the entire 96-gene panel for differences between these populations.

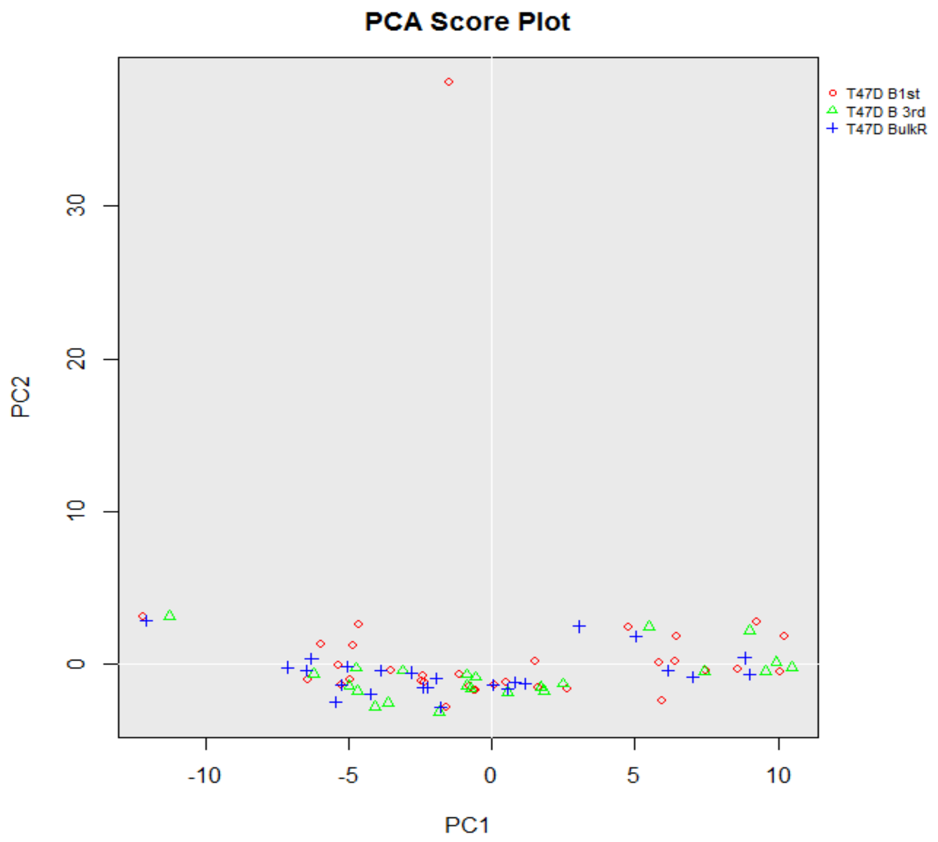

Supplementary Figure 14. Principal component analysis (PCA) plot showing log2 gene expression data of single cells from T47D breast cancer cell line. T47D B1st (Red) and T47D BulkR (Blue) are the same single cells isolated by C1 and analyzed by BioMark HD in 2 separate RT-qPCR experiments as technical replicates. T47D B3rd (Green) and T47D B1st (Red) are biological replicates of 2 independent C1/BioMark HD experiments.

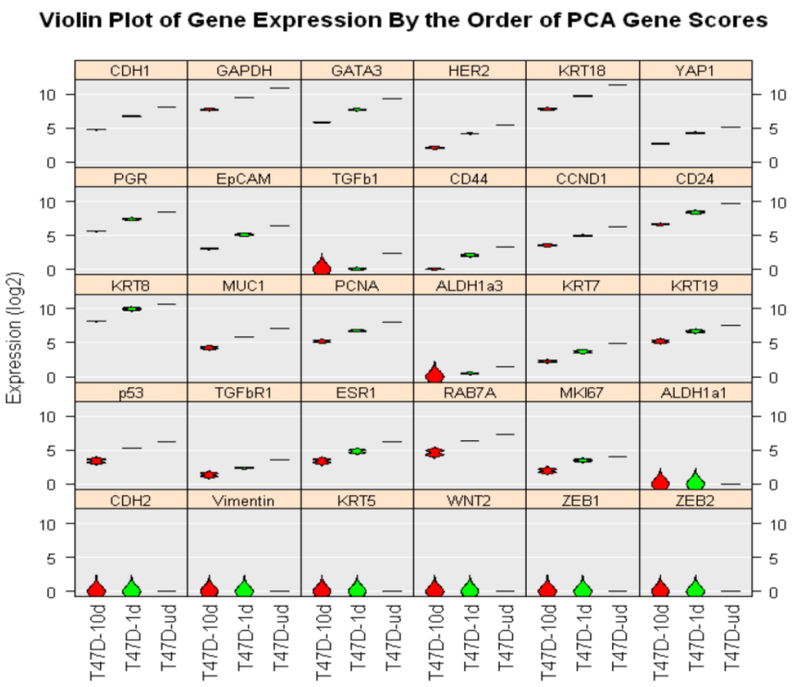

Supplementary Figure 15. Violin plot of the gene expression results of undiluted (ud), 1:1 diluted (1d), and 1:10 diluted (10d) total RNA extracted from T47D breast cancer cell line using BioMark HD system and TaqMan assays.

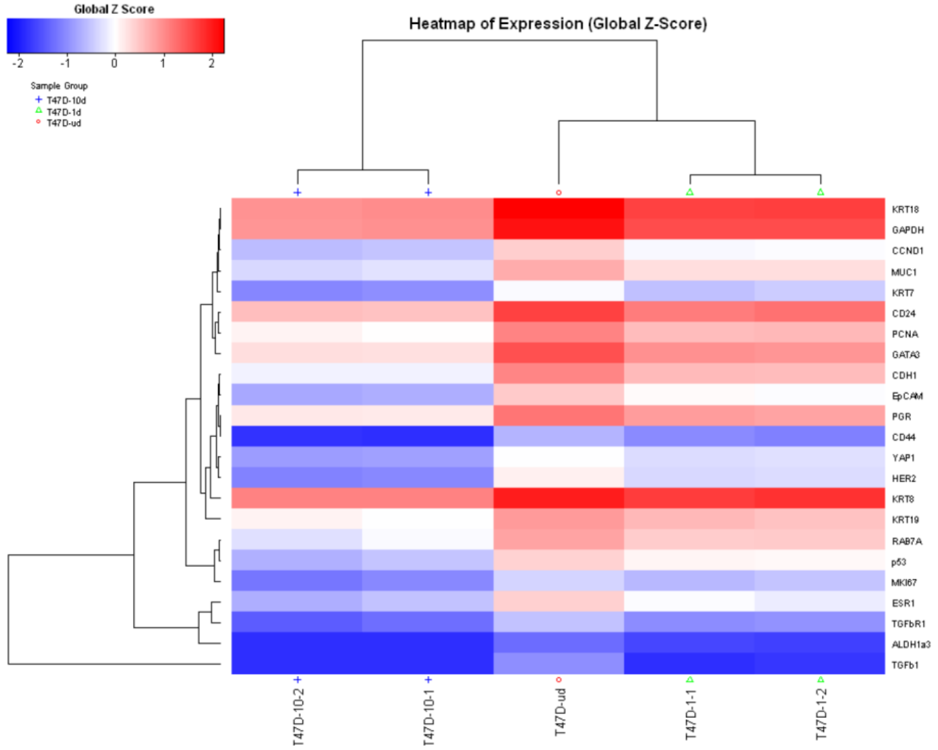

Supplementary Figure 16. Heatmap of the gene expression results of undiluted (ud), 1:1 diluted (1d), and 1:10 diluted (10d) total RNA extracted from T47D breast cancer cell line using BioMark HD system and TaqMan assays.

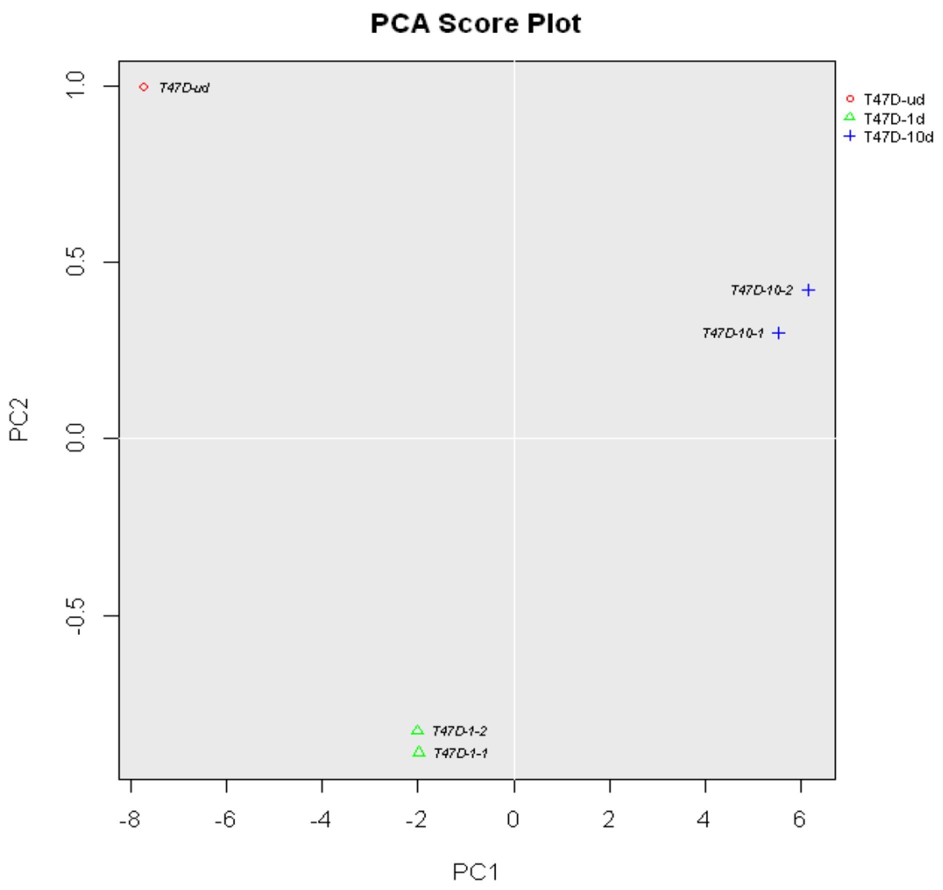

Supplementary Figure 17. PCA of the gene expression results of undiluted (ud), 1:1 diluted (1d), and 1:10 diluted (10d) total RNA extracted from T47D breast cancer cell line using BioMark HD system and TaqMan assays.

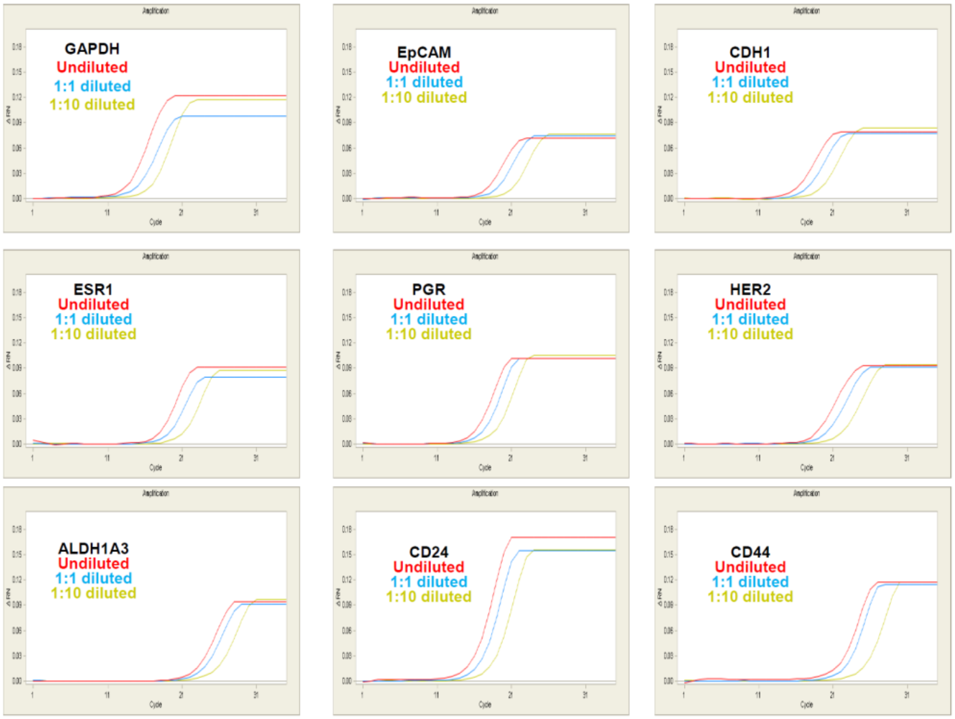

Supplementary Figure 18. Amplification plots of the critical genes of undiluted (ud), 1:1 diluted (1d), and 1:10 diluted (10d) total RNA extracted from T47D breast cancer cell line using BioMark HD system and TaqMan assays.

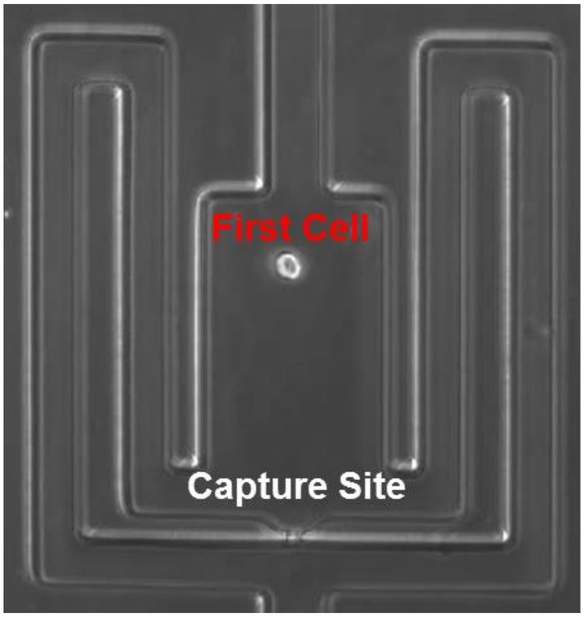

Supplementary Movie 1. A MDA-MB-231 cell was loaded into the chamber by gravity flow, and the first coming cell was captured at the capture site.

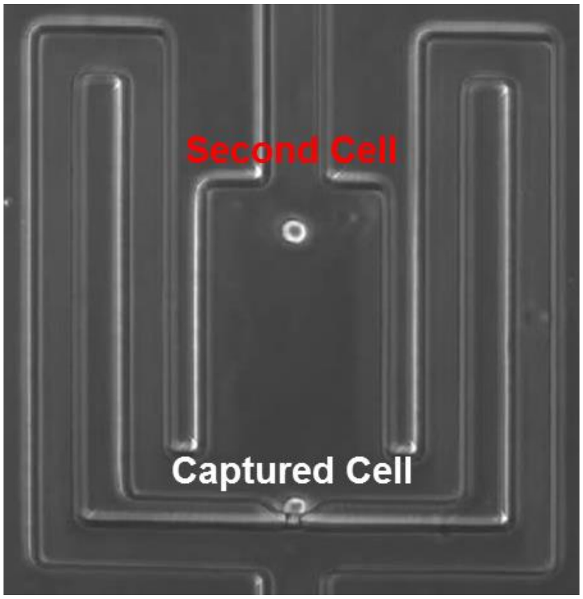

Supplementary Movie 2. A MDA-MB-231 cell was loaded into the chamber by gravity flow. As the capture site was occupied by a captured cell, and the next coming cell flow to downstream through serpentine path.
